# Personalized prediction of lymph node metastasis in papillary thyroid microcarcinoma: a nomogram and web calculator

**DOI:** 10.1038/s41598-025-28483-8

**Published:** 2025-11-25

**Authors:** Wei Zhang, Jichao Zhu, Ying Zhang, Li Sun, Kun Wang, Ying Dong, Wenhui Yan, Xiao Yu, Yidan Zhang, Wei Jia, Weiwei Wang, Anquan Shang

**Affiliations:** 1 Department of Laboratory Medicine , Affiliated Lianyungang Clinical College of Nantong University , Lianyungang, Jiangsu Province, 222006 China; 2 Department of Laboratory Medicine , Huzhou Central Hospital , Zhejiang Province, Huzhou, 313000 China; 3 School of Medicine , Xiangyang Polytechnic , Xiangyang, Hubei Province, 441021 China; 4 Clinical College of Ningxia Medical University , Yinchuan, Ningxia, 750003 China; 5 Department of Otolaryngology-Head and Neck Surgery , Shanghai Sixth People’s Hospital Shanghai Jiao Tong University School of Medicine , Shanghai, 200030 China; 6 Department of Respiratory and Critical Care Medicine , Shanghai Chest Hospital Shanghai Jiao Tong University School of Medicine , Shanghai, 200030 China; 7 Department of Pathology , The Sixth People’s Hospital of Yancheng , Yancheng, Jiangsu Province, 224001 China; 8 Department of Medical Laboratory, The Second People’s Hospital of Lianyungang, No. 41, Hailian East Road, Haizhou District, Lianyungang, Jiangsu Province, 222006 China

**Keywords:** PTMC, Lymph node metastasis, Nomogram, Prediction model, Biomarkers, Cancer, Computational biology and bioinformatics, Oncology, Risk factors

## Abstract

To analyze clinical and laboratory data of patients, identify indicators associated with lymph node metastasis (LNM) in papillary thyroid microcarcinoma (PTMC), and develop a nomogram and web-based calculator for predicting LNM risk. A retrospective analysis was performed on 754 patients who underwent PTMC resection between January 2018 and July 2023. Patients were randomly divided into a modeling set and a validation set at a 7:3 ratio. Independent predictive factors for LNM were identified using LASSO-logistic regression, and a nomogram was constructed. Model discrimination and calibration were assessed using ROC curves, calibration plots, the C-index, and the Hosmer–Lemeshow test. The modeling set included 528 cases (392 non-metastatic, 136 metastatic), and the validation set comprised 226 cases (175 non-metastatic, 51 metastatic). LASSO-logistic regression identified nodule diameter, gender, albumin, apolipoprotein B, and thyroglobulin as independent predictors of LNM. The predictive model achieved AUROC values of 0.758 in the modeling set and 0.696 in the validation set. Calibration plots, the C-index, and the Hosmer–Lemeshow test demonstrated good agreement between predicted and observed risks. Decision curve analysis (DCA) and clinical impact curves (CIC) indicated favorable clinical benefit and impact. The model was also implemented as a freely accessible web-based calculator (https://ley120.shinyapps.io/Lymph_Node_Metastasis_in_PTMC/). This study developed a nomogram and web-based calculator to predict LNM risk in PTMC. The model may assist clinicians in estimating LNM risk by entering relevant patient variables. This study was registered in the Chinese Clinical Trial Registry (ChiCTR2400080625).

**Supplementary Information:**

The online version contains supplementary material available at 10.1038/s41598-025-28483-8.

## Introduction

Thyroid cancer is a prevalent endocrine system malignancy, and its occurrence has surged globally in recent years due to improvements in diagnostic methods^1–3^. The most prevalent form of thyroid cancer is papillary thyroid cancer, accounting for approximately 85% of thyroid cancers^4–6^. According to the World Health Organization, a papillary thyroid carcinoma that measures less than 1 cm is termed papillary thyroid microcarcinoma (PTMC)^7–9^. PTMC accounts for approximately 50% of papillary thyroid carcinomas^10,11^. Although most patients with PTMC have a slow disease course and a good long-term prognosis, the some PTMC are aggressive and have the ability to metastasize locally and distantly, and even have disease-specific mortality^12–14^. Studies have shown that around 30%−60% of PTMC patients might experience lymph node metastasis(LNM)^15–17^. Surgery is the first choice for PTMC treatment and is considered overtreatment for patients with low-risk PTMC because surgery includes disadvantages such as high invasiveness, high risk of complications and the need for long-term medication^18^. The latest American Thyroid Association guidelines now recommend active surveillance management as an alternative to surgery for patients with very low-risk PTMC^19^. Therefore, identifying the risk of LNM in PTMC patients is critical in patient treatment planning and prognosis of disease progression.

Fine needle aspiration cytology(FNAC) is the most precise and economical technique for evaluating thyroid nodules. Nodules with a maximum size exceeding 1 cm and visible on ultrasound are recommended, but cytological findings could not be determined for nodules smaller than 1 cm in up to 60% of cases^20^. Ultrasound is one of the main methods for diagnosing palpable or non-palpable thyroid nodules, with the advantages of high resolution, low cost and no radiation^21,22^. Ultrasound is frequently employed as a screening method for thyroid cancer patients, but even though it plays a crucial role in diagnosing lymph node metastases, neck ultrasound has low sensitivity for detecting metastatic lesions in lymph nodes^23,24^, Reports indicate that preoperative ultrasound has a sensitivity of 23% to 55% for diagnosing lymph node metastases, meaning almost half of the patients with metastases are not diagnosed in a timely manner^25^. Furthermore, the sensitivity of CT and MRI for detecting lymph node metastases is approximately 30%^26^. Even though previous research has provided predictive models for LNM in PTMC, these studies have particular limitations. For example, some studies^27,28^ are based on proteomics to construct predictive models for LNM, Some studies^29,30^ have also focused on using radiomics to construct models that predict LNM. However, due to the fact that proteomics and radiomics are not routinely conducted in most medical institutions, the clinical applicability of these models is limited. The research by Wang et al.^31^ focused on developing a model to predict LNM risk based on pathological and ultrasonic features, however, pathological results are usually obtained only post-operation, which does not assist in the decision-making for intraoperative lymphadenectomy, thus limiting clinical application. In previous studies, there are few predictive models that combine hematological indicators with radiographic indices.

In this study, we propose to screen patients for common clinical blood indicators and analyze them in combination with clinical examination indicators. The purpose of our work is to explore the diagnostic value of both single and combined blood indicators in identifying LNM risk in PTMC patients, and to assess the clinical utility of each indicator, and create a nomogram and online calculator to estimate the likelihood of LNM in PTMC. This might serve as a reference for evaluating the risk of PTMC spreading to lymph nodes.

## Methods

### Medical records

This study is a single-center retrospective analysis of 754 patients who underwent PTMC resection in the Department of Thyroid Surgery at Huzhou Central Hospital from 2018 to 2023. 567 patients with PTMC confined to the primary site were considered as no metastatic group. 187 patients with PTMC involving cervical lymph nodes were considered as metastatic and were set as metastatic group. Thyroid imaging reporting and data system (TI-RADS) results of patients’ preoperative TI-RADS were graded and evaluated according to TI-RADS classification standard issued by American Radiological Society (ACR) in 2017^32^. This study follows the TRIPOD + AI^33^ checklist to report results.

### Inclusion and exclusion criteria

Inclusion criteria: (1) Ultrasonography was performed before operation, and TI-RADS classification was used for all nodules. (2) All samples were pathologically diagnosed as PTMC. (3) Postoperative pathology confirmed that PTMC was confined to the primary site or had metastasized to the central cervical lymph nodes. (4) According to the ultrasound, the nodule’s maximum diameter did not exceed 1 cm. (5) The patient has no history of systemic diseases such as diabetes or hypertension. (6) No other primary tumors were found. (7) The patient had no thyroid related diseases, such as hyperthyroidism, hypothyroidism or thyroiditis. (8) All individuals had lymph nodes removed for either prevention or treatment. Postoperative pathology confirmed the diagnosis of PTMC at the primary site or in cervical LNM. (9) Subcentimetric TI-RADS 1–4 nodules were included for surgery only when high-risk features were present or when patients had a strong preference for surgical treatment.

Exclusion criteria: (1) Patients with chronic inflammation or infectious diseases. (2) The pathological type was not PTMC or distant metastasis. (3) There were primary tumors in other parts. (4) Patients with serious diseases in cardiovascular, cerebrovascular, liver, kidney and other systems. (5) Patients lacking clinical and laboratory information. (6) Patients with blood disease. (7) Patients with immune deficiency. (8) History of previous thyroid surgery.

### Variables selection

Candidate blood indicators were selected based on two criteria: (1) clinical availability and routine practice. (2) previously reported relevance to disease occurrence, progression, or prognosis. Routine preoperative tests, including complete blood count, serum biochemistry, lipid profiles, and thyroid function tests, were available for all patients and thus included as candidate predictors. In addition, tumor markers such as thyroglobulin, although not universally tested, are commonly measured in patients with suspected malignant thyroid nodules and were therefore considered clinically meaningful. To reduce multicollinearity and avoid overfitting, the least absolute shrinkage and selection operator (LASSO) regression was applied to identify the most informative subset of variables for model construction. The surgical indications for sub-centimetric nodules were determined based on individual patient characteristics, risk factors, and patient preferences, rather than employing a routine total thyroidectomy for all cases.

### Clinical information and patient examination

Sixth-six patients clinical and laboratory data were collected, including nodule number, nodule diameter, LNM or not, pathological findings, white blood cells (WBC), absolute neutrophil value (NE#), absolute lymphocyte value (LY#), absolute monocyte value (MO#), red blood cells (RBC), hemoglobin (HGB), neutrophil percentage (NE%), lymphocyte percentage (LY%), monocyte percentage (MO%), ultrasound TI-RADS classification, age, gender, mean red blood cell volume (MCV), mean red blood cell hemoglobin content (MCH), mean red blood cell hemoglobin concentration (MCHC), platelets (PLT), urea nitrogen (BUN), aspartate aminotransferase (AST), albumin (ALB), globulin (GLOB), total protein (TP), blood glucose (GLU), creatinine (CRE), uric acid (UA), alanine aminotransferase (ALT), cancer antigen 199(CA199), alkaline phosphatase (ALP), direct bilirubin (DBIL), total bilirubin (TBIL), A-fetoprotein(AFP), glutamyl transpeptidase (GGT), fibronectin(FN), cholesterol (CHO), sialic acid (SA), cholinesterase(CHE), high-density lipoprotein(HDL), Adenosine deaminase(ADA), carcinoembryonic antigen(CEA), leucine aminopeptidase(LAP), triglycerides (TRIG), prothrombin time (PT), low-density lipoprotein(LDL), Apolipoprotein A-I(APOA1), international normalized ratio (INR), Apolipoprotein B(APOB), free fatty acid(FFA), Apolipoprotein E(APOE), Lipoprotein a(LPa), activated partial thromboplastin time (APTT), triiodothyronine (T3), calcitonin (CT), fibrinogen (FIB), prothrombin time (TT), D-Dimer (DD), thyroxine (T4), thyroid stimulating hormone (TSH), parathyroid hormone(PTH), thyroglobulin antibody (TGAB), free triiodothyronine (FT3), free thyroxine (FT4), thyroid peroxidase antibody (TPO), thyroglobulin (TG). The included blood tests were based on the patient’s first empty blood sample collected within 24 h after the consultation. In this study, some of the test items (such as complete blood count, biochemical indicators, blood lipids, and thyroid function) are part of the routine preoperative examinations in our hospital. All enrolled patients have complete data, and the testing costs are relatively low and generally acceptable to patients. Certain tumor markers, such as thyroglobulin, are commonly used in clinical practice for the routine evaluation of patients with suspected malignant thyroid nodules, and are therefore more prevalent and accessible. The indicators included in this study were all derived from routine clinical practices or commonly used examinations, ensuring that the model construction is based on actual clinical scenarios. This investigation was conducted in accordance with the Declaration of Helsinki’s guiding principles and was sanctioned by the Medical Ethics Committee (202207002-01). This research involves a retrospective analysis. Since it doesn’t involve recruiting patients or gathering personal data, written informed consent from participants is not required. This study was registered in the Chinese Clinical Trial Registry (ChiCTR2400080625).

### Testing instruments and indicators assignment

TI-RADS classification was reported by 4D ultrasound using PHILIPS HD11XE; sysmex xn9000 and its supporting reagents were to detect WBC, NE#, NE%, LY#, LY%, MO#, MO%, RBC, HGB, MCV, MCH, MCHC, PLT, CRP; Abbott c16000 and its supporting reagents were to detect GLU, BUN, CRE, UA, AST, ALT, TP, ALB, TBIL, DBIL, ALP, GGT, SA, FN, CHE, ADA, LAP, TRIG, CHO, HDL, LDL, APOA1, APOB, AOPE, FFA, LPa; WERFEN ACLTOP750 and its companion reagents were used for PT, APTT, FIB, TT, DD; Abbott i4000 and its companion reagents for T3, T4, TSH, FT3, FT4, TPO, TGAB, TG, CT, AFP, CEA, CA199, PTH;

### Sample size calculation

As per the generally accepted technique for sample size calculation in statistics, we calculated the sample size using the event per variable (EPV) indicator for each variable^34^. The likelihood of PTMC cases involving cervical LNM in our data is 0.25. Given that we calculated the inclusion of five predictor variables and set the EPV to 10, we used the following formula for the calculation:

According to the formula, the modeling set needs to include at least 200 patients. By enlarging the sample size, the accuracy and reliability of the predictive model can be enhanced, and the sample size satisfies the necessary conditions.

### Statistical analysis

SPSS 25.0 was employed to perform statistical analysis on the qualifying data, with the Shapiro-Wilk test used to assess the normality of the measurement data. In this study, the proportion of missing values for the main variables included in the analysis was less than 5%, and missing data were imputed using the mean for normally distributed data or the median for non-normally distributed data. The normally distributed data were expressed by (x ± SD) and two independent samples t-test was used for comparison between groups of the data. The χ^2^ test was used for counting data. The median (M) and percentile (P25, P75) were used for the skewed distribution of the measurement data and the Mann-Whitney U test was used for the comparison between groups of the data. Spearman’s rank correlation analysis was employed to analyze the correlation of each indicator with common discriminatory indicators. Generate ROC curves to assess the sensitivity, specificity, ideal cutoff point, Youden index, negative predictive value (NPV), and positive predictive value (PPV) for each variable. Use the area under the curve (AUROC) to determine classification performance, and compare the AUROC of each indicator using the Delong test. Precision-recall curves (PRC) and area under PRC (AUPRC) for each indicator were drawn by medcalc software to prevent the ROC curve evaluation of discrimination insensitivity when the proportion of positive and negative samples is extremely unbalanced. Decision curve analysis (DCA) and clinical impact curves (CIC) were used to evaluate the net clinical benefit of nodal diameter, gender, ALB, APOB, TG and the five indicators combination. The model’s net clinical benefit rate was assessed by the net benefit across various threshold probabilities, It was computed by taking the difference between the proportion of true-positive and false-positive patients, while also weighing the potential harm of not performing the intervention against the negative impact of performing it unnecessarily. The enrolled patients were randomly divided into a modeling set and a validation set in a 7:3 ratio.The final LASSO regression, Harrell’s C-index, Hosmer-Lemeshow test, calibration curve, DCA and CIC were performed. LASSO regression for age, gender, ultrasound TI-RADS classification, Nodal diameter, number of nodules and laboratory test indicators were screened and downscaled, identified were the variables with regression coefficients that were non-zero. The meaningful variables screened by LASSO regression analysis were subjected to multicollinearity diagnosis, and the relevant indicators without multicollinearity were included in the multivariable logistic regression analysis to identify the independent influencing factors for LNM in PTMC patients. Based on nodal diameter, gender, ALB, APOB, and TG, a nomogram plot was developed to estimate the risk of LNM in PTMC patients. The model’s performance was assessed through discrimination and calibration, with AUROC used to evaluate discrimination. Meanwhile, a 10-fold cross-validation was performed on the modeling set to calculate and report the overall and average AUC along with the 95% CI. The calibration degree of a prediction model describes the match between the predicted probability and the actual observed value, The calibration curve, along with the C-index calculation and Hosmer-Lemeshow test, demonstrates the calibration degree. The calibration curve was validated using the Bootstrap method with 1000 internal repeated samplings. The AUROC and C-index values are between 0.5 and 1. The closer to 1 indicates the better discriminative differentiation efficiency and consistency of the prediction model. To facilitate practical application, the model was deployed on a web application platform based on shinyapp. In this study, glmnet, rms, foreign, rmda, survival, ggplot2, regplot, tidyverse, and caret packages in R software 4.3.2 were used, and differences were considered statistically significant at *p <* 0.05.

## Results

### Baseline information

The enrolled patients were randomly split into the modeling set and the validation set according to the ratio of 7:3. There were 528 patients in the modeling set, including 392 patients in the non-metastatic group and 136 patients in the metastatic group. A total of 226 patients were in the validation set, comprising 175 non-metastatic and 51 metastatic patients.

The comparison of enrolled patients information showed that there was no statistically significant difference between the modeling set and the validation set in cervical LNM, age, node number, TI-RADS classification, nodal diameter, WBC, ALT, AST, GLU, BUN, CRE, AFP, CEA, etc. (*p >* 0.05). Statistical significance (*p* < 0.05) was found between the modeling set and the validation set only for gender, RBC, and HGB. If the model established in the modeling set obtains favorable predictive value in the validation set, it will further prove the positive applicability of the model. The comparison of clinical data of patients in modeling set and validation set is shown in Table 1.

**Table 1:** The comparison of clinical data between patients in modeling set and validation set.

Indicator	Modeling set(*n* = 528)	Validation set(*n* = 226)	Statistics	*P*
Cervical lymph node			0.864	0.353
metastasis	392	175		
Non-metastasis	136	51		
TI-RADS classification			5.688	0.224
1–3	8	1		
4a	97	38		
4b	171	63		
4c	81	47		
5–6	171	77		
Nodule number			1.451	0.484
1	150	59		
2	111	42		
≥ 3	267	125		
Gender			3.874	0.049
Female	395	184		
Male	133	42		
Nodal diameter(CM)	0.6(0.5,0.8)	0.6(0.5,0.8)	−0.359	0.719
Age(years)	46(35,53)	46(35,53)	−0.218	0.828
WBC(×10^9^/L)	5.3(4.5,6.4)	5.4(4.6,6.5)	−0.919	0.358
NE#(×10^9^/L)	3.2(2.6,4.0)	3.2(2.6,4.0)	−0.711	0.477
NE(%)	60.48 ± 8.54	60.49 ± 8.29	−0.014	0.989
LY#(×10^9^/L)	1.6(1.3,2.0)	1.7(1.3,2.0)	−1.419	0.156
LY(%)	30.67 ± 7.56	30.88 ± 7.66	−0.337	0.736
MO#(×10^9^/L)	0.3(0.3,0.4)	0.3(0.3,0.4)	−1.531	0.126
MO(%)	6.1(5.3,7.2)	6.3(5.2,7.3)	−0.590	0.555
RBC(×10^12^/L)	4.64(4.35,4.94)	4.55(4.32,4.91)	−2.169	0.030
HGB(g/L)	137(129,148)	135(126,144)	−2.049	0.040
MCV(fL)	89.8(87.0,92.4)	89.9(87.5,92.3)	−0.703	0.482
MCH(pg)	29.7(28.8,30.8)	29.8(28.8,30.8)	−0.458	0.647
MCHC(g/L)	331(324,338)	330(324,336)	−0.790	0.430
PLT (×10^9^/L)	210(179,257)	219(184,260)	−1.033	0.301
GLU(mmol/L)	5.25(4.92,5.61)	5.15(4.87,5.52)	−1.674	0.094
BUN(mmol/L)	4.6(3.8,5.5)	4.5(3.7,5.4)	−1.040	0.298
CRE(µmol/L)	63.4(58.3,71.8)	62.0(58.2,68.1)	−1.736	0.083
UA(µmol/L)	302.3(257.4,355.1)	306.8(252.2,350.0)	−0.267	0.789
AST(U/L)	18.9(17.1,21.2)	18.6(16.8,21.6)	−1.392	0.164
ALT(U/L)	15.3(12.0,24.2)	15.3(12.3,21.3)	−0.513	0.608
TP(g/L)	74.90 ± 4.30	74.58 ± 4.21	0.955	0.340
ALB(g/L)	44.31 ± 2.48	44.11 ± 2.41	0.996	0.320
GLOB(g/L)	30.5(28.3,32.7)	29.9(28.1,33.1)	−0.847	0.397
TBIL (µmol/L)	12.0(9.4,16.3)	12.1(9.7,15.5)	−0.027	0.979
DBIL(µmol/L)	4.6(3.7,5.9)	4.8(3.8,5.9)	−0.514	0.607
ALP(U/L)	69.1(56.0,84.8)	68.7(51.8,82.7)	−1.324	0.185
GGT(U/L)	15.4(10.9,28.0)	14.5(10.4,24.5)	−1.698	0.089
SA(mg/dL)	56.9(52.5,61.5)	57.2(52.6,61.1)	−0.161	0.872
FN(mg/L)	249.5(230.1,275.0)	249.8(232.9,268.8)	−0.250	0.803
CHE(U/L)	10,350(9130,11766)	10,311(9055,11717)	−0.420	0.674
ADA(U/L)	9.0(7.6,10.7)	8.8(7.5,10.5)	−1.029	0.304
LAP(U/L)	22.6(20.3,25.6)	21.8(20.0,25.4)	−1.458	0.145
TRIG(mmol/L)	1.18(0.85,1.79)	1.23(0.82,1.82)	−0.030	0.976
CHO(mmol/L)	4.64(4.16,5.24)	4.62(4.10,5.11)	−0.933	0.351
HDL(mg/dL)	50.10(42.97,59.30)	49.26(43.63,56.90)	−0.623	0.533
LDL(mg/dL)	103.04(84.81,120.41)	101.72(85.86,120.77)	−0.061	0.951
APOA1(g/L)	1.50(1.35,1.67)	1.49(1.34,1.65)	−1.012	0.312
APOB(g/L)	0.91(0.79,1.04)	0.90(0.79,1.02)	−0.701	0.483
APOE(mg/L)	40.9(34.8,49.8)	40.6(34.1,48.9)	−0.831	0.406
FFA(µmol/L)	489.0(365.3,624.6)	482.4(365.2,628.5)	−0.364	0.716
LPa(mg/dL)	10.8(6.4,21.5)	10.9(6.8,19.2)	−0.118	0.906
PT(s)	11.4(11.0,11.8)	11.4(11.0,11.8)	−0.218	0.827
INR	0.96(0.93,0.99)	0.97(0.94,1.00)	−1.332	0.183
APTT(s)	29.9(28.0,32.0)	29.3(27.9,31.5)	−1.504	0.133
FIB(s)	2.70(2.36,2.96)	2.72(2.37,3.00)	−0.640	0.522
TT(s)	15.6(14.7,17.3)	15.8(14.7,18.6)	−1.645	0.100
DD(mg/L)	0.2(0.14,0.29)	0.21(0.14,0.32)	−0.929	0.353
T3(ng/mL)	1.01(0.91,1.11)	1.01(0.91,1.10)	−0.439	0.661
T4(µg/dL)	7.75(7.03,8.79)	7.81(7.01,8.60)	−0.204	0.839
TSH(uIU/mL)	1.650(1.113,2.225)	1.655(1.153,2.466)	−0.635	0.526
FT3(pg/mL)	3.05(2.83,3.27)	3.08(2.80,3.26)	−0.168	0.866
FT4(ng/dL)	1.01(0.94,1.08)	1.00(0.95,1.07)	−0.384	0.701
TGAB(IU/mL)	1.21(0.74,4.24)	1.10(0.76,6.89)	−0.027	0.978
TPO(IU/mL)	0.48(0.27,1.37)	0.49(0.27,1.42)	−0.003	0.998
TG(µg/L)	9.32(4.95,17.13)	10.34(5.01,21.49)	−0.834	0.404
CT(ng/mL)	0.7(0.5,1.8)	0.7(0.5,1.7)	−0.509	0.611
PTH(pg/mL)	42.0(31.9,53.5)	44.5(33.4,53.8)	−0.801	0.423
CRP(mg/L)	0.5(0.5,0.5)	0.5(0.5,0.5)	−0.804	0.421
AFP(ng/mL)	2.5(1.87,3.55)	2.66(1.85,3.57)	−0.632	0.527
CEA(ng/mL)	1.37(0.97,2.11)	1.35(0.86,2.00)	−1.287	0.198
CA199(U/mL)	5.41(2.82,11.28)	5.77(2.95,9.48)	−0.711	0.477

### Comparison of clinical data between patients in the metastasis group and those in the non-metastasis group in the modeling set

The TI-RADS classification, age, gender, nodal diameter, WBC, NE#, LY#, MO#, RBC, HGB, MCHC, CRE, UA, TP, LAP, CHO, LDL, APOB, FFA, DD, TSH, FT4, TGAB, TG, and CT of patients in both groups were compared, Statistical significance was observed in the differences, with *p* < 0.05. There was no statistically significant difference between the two groups when comparing the levels of hematological indicators such as nodule number, GLU, BUN, AST, ALT, TBILL, ALP, GGT, LPa, PT, T3, T4, PTH, CRP, AFP, CEA, and CA199 (*p >* 0.05). (Table 2).

**Table 2:** Comparison of clinical data between patients with and non-metastasis in the modeling set.

Indicator	Non-metastasis(*n* = 392)	metastasis(*n* = 136)	Statistics	*P*
TI-RADS classification			11.849	0.019
1–3	4	4		
4a	82	15		
4b	131	40		
4c	54	27		
5–6	121	50		
Nodule number			1.883	0.390
1	117	33		
2	83	28		
≥ 3	192	75		
Gender			29.626	0.000
Female	317	78		
Male	75	58		
Nodal diameter(CM)	0.6(0.5,0.8)	0.7(0.5,0.9)	−4.630	0.000
Age(years)	48(36,54)	42(33,48)	−3.723	0.000
WBC(×10^9^/L)	5.2(4.4,6.3)	5.7(4.6,7.0)	−2.964	0.003
NE#(×10^9^/L)	3.1(2.6,3.8)	3.4(2.7,4.2)	−2.094	0.036
NE(%)	60.69 ± 8.64	59.88 ± 8.27	0.949	0.343
LY#(×10^9^/L)	1.5(1.3,1.9)	1.7(1.3,2.1)	−2.837	0.005
LY(%)	30.61 ± 7.60	30.86 ± 7.48	−0.331	0.740
MO#(×10^9^/L)	0.3(0.3,0.4)	0.3(0.3,0.4)	−2.805	0.005
MO(%)	6.1(5.2,7.2)	6.1(5.4,7.1)	−0.910	0.363
RBC(×10^12^/L)	4.61(4.35,4.89)	4.72(4.42,5.12)	−2.662	0.008
HGB(g/L)	135(128,144)	141(131,155)	−3.765	0.000
MCV(fL)	89.7(86.93,92.2)	90.0(87.05,92.6)	−0.547	0.584
MCH(pg)	29.7(28.7,30.7)	29.9(29.0,30.98)	−1.447	0.148
MCHC(g/L)	330(323,337)	333(326,339)	−2.359	0.018
PLT (×10^9^/L)	210(177,256)	216(180,261)	−0.896	0.370
GLU(mmol/L)	5.25(4.93,5.58)	5.25(4.87,5.72)	−0.223	0.823
BUN(mmol/L)	4.5(3.8,5.4)	4.6(3.8,5.5)	−0.176	0.860
CRE(µmol/L)	62.8(57.9,70.0)	65.5(59.8,75.1)	−3.142	0.002
UA(µmol/L)	299.2(253.2,351.9)	321.4(267.8,368.7)	−2.270	0.023
AST(U/L)	18.9(17.1,21.1)	18.7(17.3,21.3)	−0.302	0.762
ALT(U/L)	15.1(11.9,23.3)	16.1(12.5,27.1)	−1.642	0.101
TP(g/L)	75.14 ± 4.11	74.21 ± 4.77	2.195	0.029
ALB(g/L)	44.39 ± 2.35	44.07 ± 2.81	1.172	0.243
GLOB(g/L)	30.76 ± 3.33	30.13 ± 3.72	1.818	0.070
TBIL (µmol/L)	11.8(9.3,16.0)	12.5(9.8,16.9)	−1.000	0.317
DBIL(µmol/L)	4.6(3.7,5.8)	4.6(3.9,6.4)	−1.197	0.231
ALP(U/L)	69.2(57.5,85.6)	67.9(55.1,81.6)	−1.499	0.134
GGT(U/L)	15.1(10.6,26.6)	16.5(11.1,31.2)	−1.301	0.193
SA(mg/dL)	57.1(52.4,61.9)	56.6(52.5,61.1)	−0.665	0.506
FN(mg/L)	250.6(230.9,276.7)	247.4(228.1,269.5)	−0.961	0.336
CHE(U/L)	10,363(9128,11774)	10,207(9150,11755)	−0.245	0.806
ADA(U/L)	9.1(7.7,10.8)	8.6(7.5,10.2)	−1.890	0.059
LAP(U/L)	22.4(20.0,25.3)	23.4(20.7,26.3)	−2.114	0.035
TRIG(mmol/L)	1.2(0.86,1.81)	1.11(0.83,1.75)	−1.060	0.289
CHO(mmol/L)	4.71(4.2,5.35)	4.49(4.09,4.99)	−2.937	0.003
HDL(mg/dL)	50.57(43.57,60.11)	48.50(41.24,56.57)	−1.922	0.055
LDL(mg/dL)	104.15(85.09,123.22)	99.42(83.03,115.12)	−2.402	0.016
APOA1(g/L)	1.51(1.37,1.69)	1.46(1.33,1.67)	−1.674	0.094
APOB(g/L)	0.92(0.79,1.06)	0.90(0.77,0.99)	−2.379	0.017
APOE(mg/L)	41.15(35.13,50.23)	39.55(33.58,48.13)	−1.383	0.167
FFA(µmol/L)	502.3(370.3,634.9)	458.5(359.6,575.2)	−1.980	0.048
LPa(mg/dL)	11.6(6.5,22.2)	9.5(5.5,20.1)	−1.444	0.149
PT(s)	11.4(11.0,11.8)	11.4(11.1,11.7)	−0.578	0.563
INR	1.0(0.9,1.0)	1.0(0.9,1.0)	−0.418	0.676
APTT(s)	29.9(27.7,31.8)	30.0(28.5,32.3)	−1.130	0.259
FIB(s)	2.7(2.38,2.98)	2.69(2.36,2.9)	−0.620	0.535
TT(s)	15.7(14.8,17.3)	15.3(14.4,16.9)	−1.835	0.066
DD(mg/L)	0.2(0.15,0.31)	0.19(0.14,0.26)	−1.974	0.048
T3(ng/mL)	1.0(0.91,1.1)	1.02(0.91,1.14)	−1.333	0.183
T4(µg/dL)	7.75(7.08,8.77)	7.67(6.92,8.84)	−0.331	0.741
TSH(uIU/mL)	1.706(1.168,2.326)	1.568(1.042,2.001)	−2.424	0.015
FT3(pg/mL)	3.05(2.82,3.26)	3.05(2.87,3.28)	−0.373	0.709
FT4(ng/dL)	1.0(0.94,1.08)	1.03(0.96,1.11)	−2.052	0.040
TGAB(IU/mL)	1.32(0.78,8.25)	1.06(0.68,1.85)	−3.268	0.001
TPO(IU/mL)	0.5(0.27,1.86)	0.41(0.29,0.91)	−1.587	0.113
TG(µg/L)	8.32(4.05,14.69)	14.15(8.4,24.4)	−6.200	0.000
CT(ng/mL)	0.6(0.5,1.5)	1.3(0.5,2.6)	−4.724	0.000
PTH(pg/mL)	42.5(32.2,53.1)	40.9(30.6,55.8)	−0.614	0.539
CRP(mg/L)	0.5(0.5,0.5)	0.5(0.5,0.5)	−0.408	0.683
AFP(ng/mL)	2.51(1.87,3.62)	2.49(1.89,3.32)	−0.351	0.726
CEA(ng/mL)	1.36(0.97,2.09)	1.44(0.90,2.12)	−0.307	0.759
CA199(U/mL)	5.27(2.78,11.37)	5.91(3.26,10.62)	−0.507	0.613

### Identification of independent variables for the modeling set based on LASSO regression

**Fig. 1:**
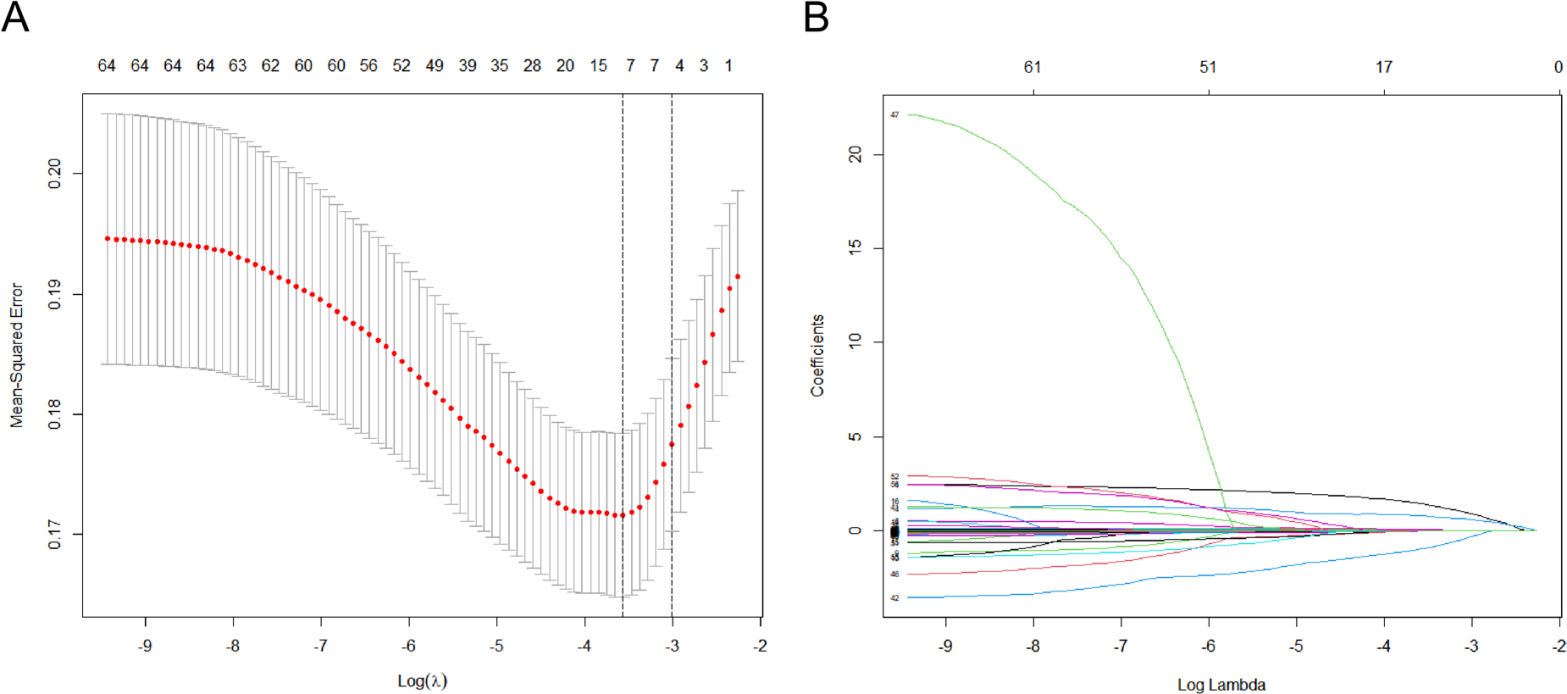
Identification of independent variables for the modeling set based on LASSO regression. **A**: Screening of optimal values of parameter λ in the LASSO model using the minimum criterion for 10-fold cross-validation; **B**: LASSO regression screening variable profiles. Nine variables with non-zero coefficients were retained (Nodule diameter, Gender, Age, WBC, ALB, APOB, TGAB, TPO, TG).

Total 65 patient-related indicators were included in this study, including age, gender, ultrasound TI-RADS classification, nodule diameter, nodule number, and laboratory hematological indicators. LASSO regression was employed to filter and reduce the 65 independent variables to pinpoint the key variables for LNM in PTMC patients. The best outcomes for the screened variables were achieved when Lambda was set to the optimal value of 0.02816074, at which time nine variables with non-zero regression coefficients were screened, namely nodal diameter, gender, age, WBC, ALB, APOB, TGAB, TPO, and TG. (Figure 1A and B).

### Identification of risk factors for LNM in PTMC patients in the modeling set using multivariable logistic regression analysis

The nine variables (nodal diameter, gender, age, WBC, ALB, APOB, TGAB, TPO, TG) identified by LASSO regression analysis were included for covariance diagnosis, and the factors showed no multicollinearity, as indicated by variance inflation factors (VIFs) of 1.069, 1.130, 1.218, 1.109, 1.082, 1.179, 1.122, 1.114, and 1.086.

Logistic regression analysis utilized the nine variables chosen through LASSO regression as independent variables. The results showed that nodule diameter, gender, ALB, APOB, and TG were independent predictive risk factors for LNM in PTMC patients, as shown in Table 3.

**Table 3:** The multivariable logistic regression of LNM in PTMC patients.

Variables	B	S.E.	Wald	*P* value	OR	95%CI	
						Lower limit	Upper limit
Nodal diameter	2.247	0.574	15.349	0.000	9.462	3.074	29.124
Gender(male)	1.155	0.255	20.606	0.000	3.175	1.928	5.229
Age	−0.019	0.011	3.212	0.073	0.981	0.960	1.002
WBC	0.144	0.077	3.453	0.063	1.155	0.992	1.344
ALB	−0.095	0.047	4.091	0.043	0.909	0.830	0.997
APOB	−1.899	0.657	8.351	0.004	0.150	0.041	0.543
TGAB	−0.005	0.003	3.166	0.075	0.995	0.989	1.001
TPO	−0.001	0.001	1.513	0.219	0.999	0.996	1.001
TG	0.014	0.006	5.891	0.015	1.014	1.003	1.025

### Correlation between nodal diameter, ALB, APOB, TG levels and common markers of thyroid tumor in two groups of patients

#### Correlation between nodal diameter and common markers of thyroid tumor

In the non-metastatic group of patients, there was a positive correlation between nodule diameter and TG levels (*r* = 0.136, *P* = 0.007); however, no correlation was observed between nodule diameter and CEA, CT, TGAB, or TSH (*r*=−0.009, *P* = 0.862; *r*=−0.009, *P* = 0.857; *r* = 0.056, *P* = 0.268; *r*=−0.008, *P* = 0.869).

**Fig. 2:**
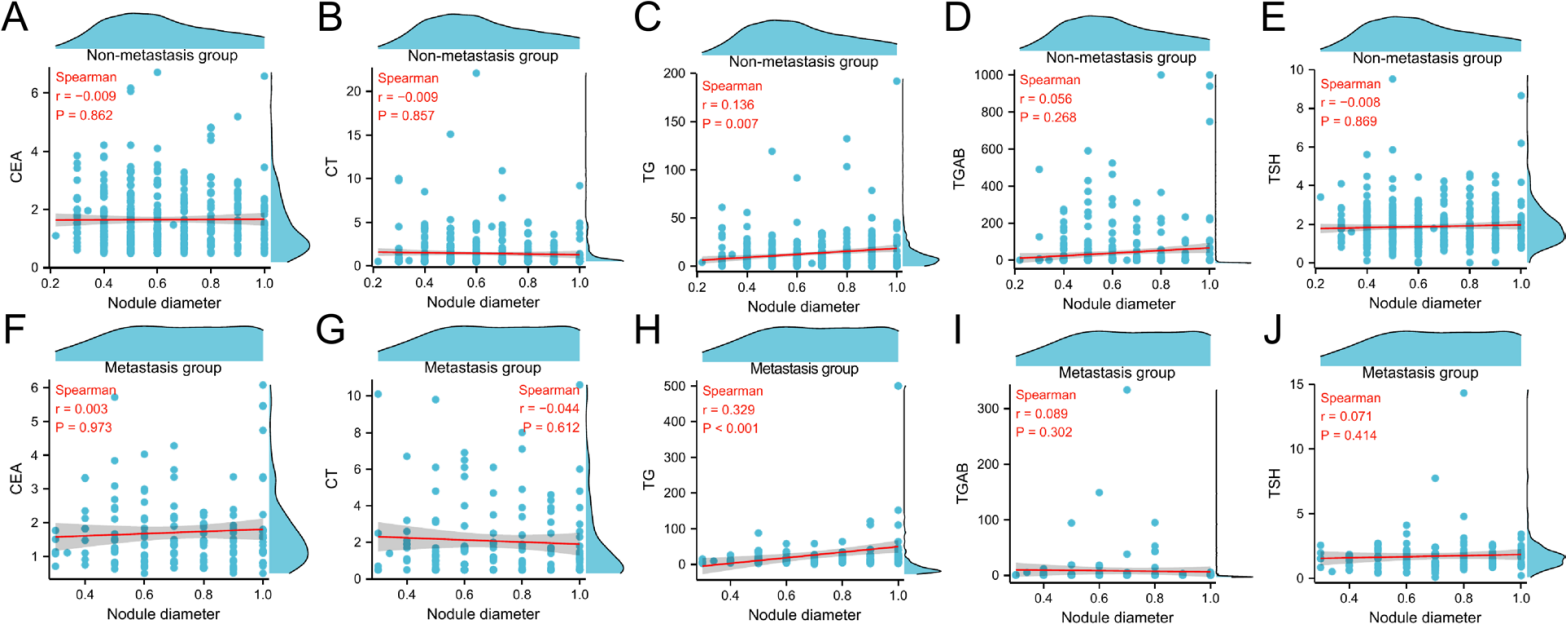
Correlation between nodal diameter and common thyroid tumor markers in two groups. **A**. Correlation between nodal diameter and CEA in non-metastatic group. **B**. Correlation between nodal diameter and CT in non-metastatic group. **C**. Correlation between nodal diameter and TG in non-metastatic group. **D**. Correlation between nodal diameter and TGAB in non-metastatic group. **E**. Correlation between nodal diameter and TSH in non-metastatic group. **F**. Correlation between nodal diameter and CEA in metastasis group. **G**. Correlation between nodal diameter and CT in metastasis group. **H**. Correlation between nodal diameter and TG in metastasis group. **I**. Correlation between nodal diameter and TGAB in metastasis group. **J**. Correlation between nodal diameter and TSH in metastasis group. nodule diameter was positively correlated with TG in both groups; no significant associations with CEA, CT, TGAB, or TSH.

In the metastatic group, nodule diameter showed a positive correlation with TG levels (*r* = 0.329, *P* < 0.001); however, there was no correlation between nodule diameter and CEA, CT, TGAB, or TSH (*r* = 0.003, *P* = 0.973; *r*=−0.044, *P* = 0.612; *r* = 0.089, *P* = 0.302; *r*=−0.071, *P* = 0.414). (Fig. 2).

#### Correlation between ALB and common markers of thyroid tumor

In the non-metastatic group of patients, ALB showed a positive correlation with CT levels (*r* = 0.125, *P* = 0.014); however, ALB exhibited no correlation with CEA, TG, TGAB, or TSH (*r* = 0.001, *P* = 0.984; *r*=−0.082, *P* = 0.103; *r*=−0.078, *P* = 0.124; *r*=−0.050, *P* = 0.321).

**Fig. 3:**
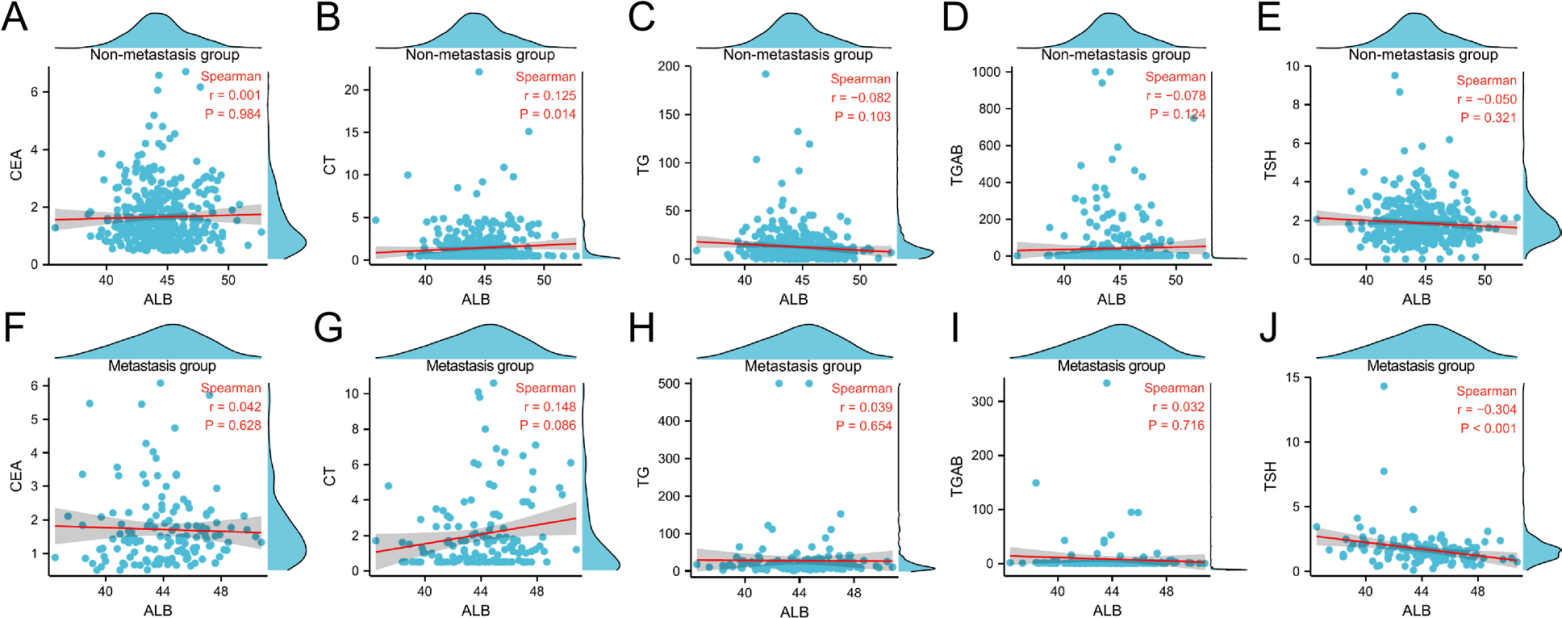
Correlation between ALB and common thyroid tumor markers in two groups. **A**. Correlation between ALB and CEA in non-metastatic group. **B**. Correlation between ALB and CT in non-metastatic group. **C**. Correlation between ALB and TG in non-metastatic group. D. Correlation between ALB and TCAB in non-metastatic group. **E**. Correlation between ALB and TSH in non-metastatic group. **F**. Correlation between ALB and CEA in metastasis group. **G**. Correlation between ALB and CT in metastasis group. **H**. Correlation between ALB and TG in metastasis group. **I**. Correlation between ALB and TGAB in metastasis group. **J**. Correlation between ALB and TSH in metastasis group. ALB correlated positively with CT in the non-metastatic group and negatively with TSH in the metastatic group.

In the metastatic group, ALB levels were negatively correlated with TSH levels (*r*=−0.304, *P* < 0.001); ALB levels showed no correlation with CEA, CT, TG, or TGAB (*r* = 0.042, *P* = 0.628; *r*=−0.148, *P* = 0.086; *r* = 0.039, *P* = 0.654; *r* = 0.032, *P* = 0.716). (Fig. 3).

#### Correlation between APOB and common markers of thyroid tumor

**Fig. 4:**
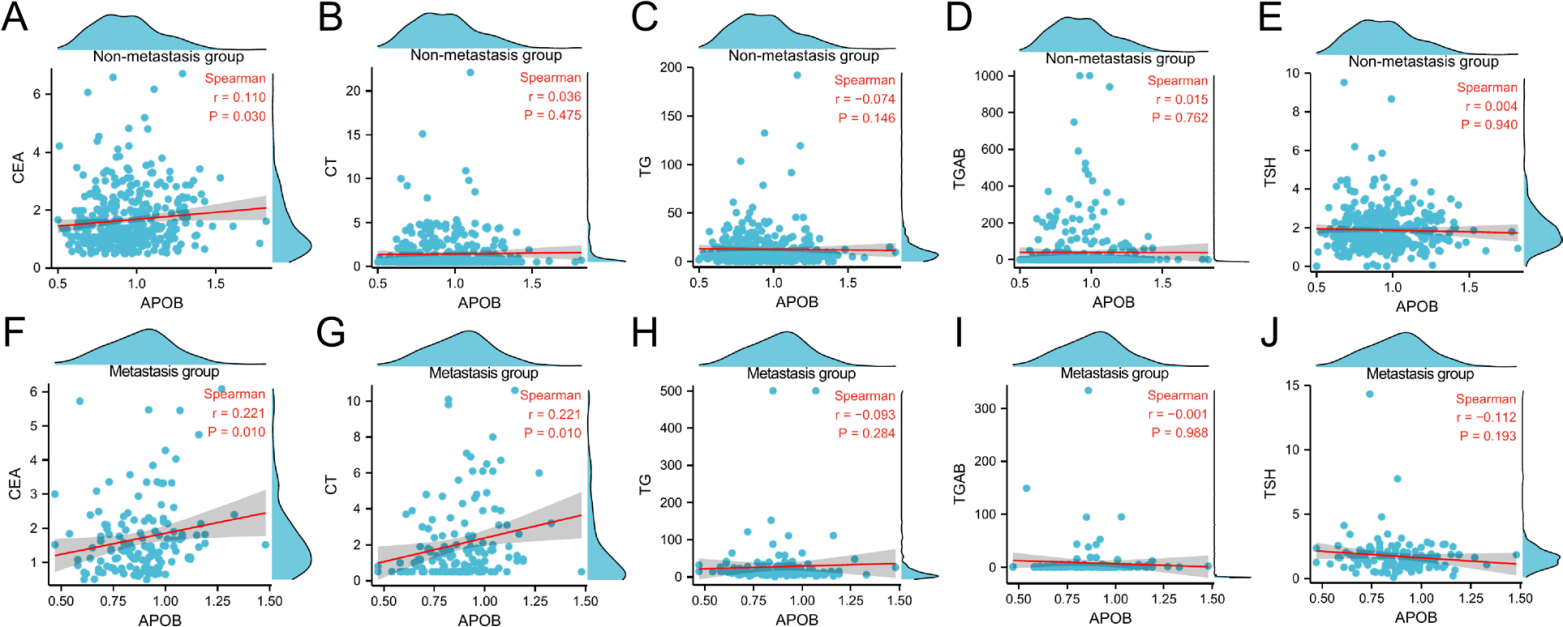
Correlation between APOB and common thyroid tumor markers in two groups. **A**. Correlation between APOB and CEA in non-metastatic group. **B**. Correlation between APOB and CT in non-metastatic group. B. Correlation between APOB and CT in non-metastatic group. **C**. Correlation between APOB and TG in non-metastatic group. **D**. Correlation between APOB and TCAB in non-metastatic group. **E**. Correlation between APOB and TSH in metastasis group. **F**. Correlation between APOB and CEA in metastasis group. **G**. Correlation between APOB and CT in metastasis group. **H**. Correlation between APOB and TG in metastasis group. **I**. Correlation between APOB and TGAB in metastasis group. **J**. Correlation between APOB and TSH in metastasis group. In metastatic patients, APOB correlated positively with CEA and CT; no significant associations with other markers.

In the non-metastatic group of patients, APOB showed a positive correlation with CEA levels (*r* = 0.110, *P* = 0.030); APOB had no correlation with CT, TG, TGAB, or TSH (*r* = 0.038, *P* = 0.475; *r*=−0.074, *P* = 0.146; *r* = 0.015, *P* = 0.762; *r*=−0.004, *P* = 0.940). In the metastatic group, APOB was positively correlated with CEA and CT levels (*r* = 0.221, *P* = 0.010; *r* = 0.221, *P* = 0.010); APOB showed no correlation with TG, TGAB, and TSH (*r*=−0.093, *P* = 0.284; *r*=−0.001, *P* = 0.988; *r*=−0.112, *P* = 0.193). (Fig. 4).

#### Correlation between TG and common markers of thyroid tumor

In the non-metastatic group of patients, TG levels were negatively correlated with TGAb levels (*r*=−0.379, *P* < 0.001); TG levels showed no correlation with CEA, CT, or TSH (*r*=−0.023, *P* = 0.652; *r*=−0.090, *P* = 0.076; *r* = 0.035, *P* = 0.490).

**Fig. 5:**
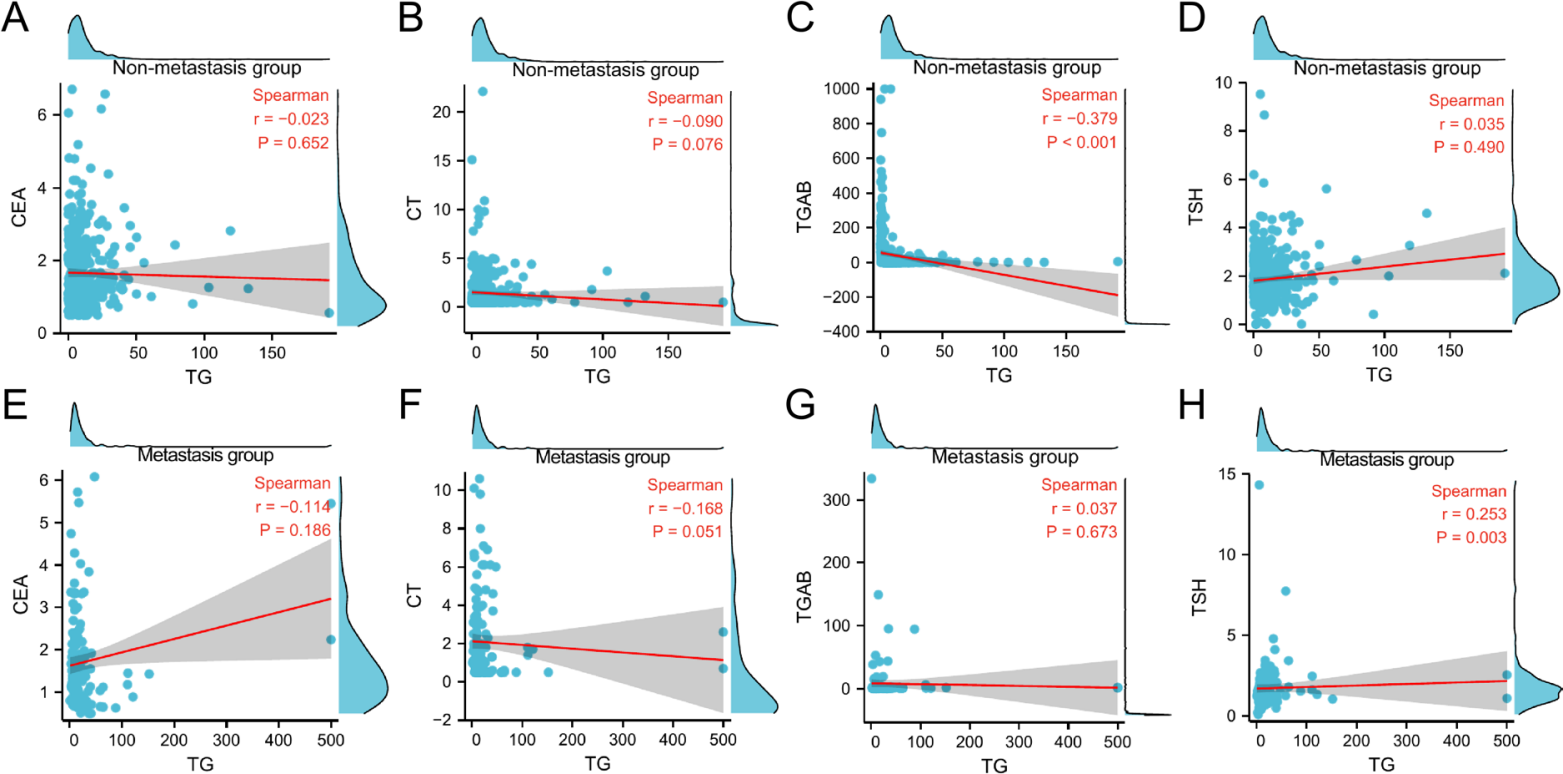
Correlation between TG and common thyroid tumor markers in two groups. **A**. Correlation between TG and CEA in the non-metastasis groups. **B**. Correlation between TG and CEA in the non-metastasis groups. **C**. Correlation between TG and CT in the non-metastasis groups. **D**. Correlation between TG and TGAB in the non-metastasis groups. **E**. Correlation between TG and CEA in the metastatic group. **F**. Correlation between TG and CT in the metastatic group. **G**. Correlation between TG and TGAB in the metastatic group. **H**. Correlation between TG and TSH in the metastatic group. TG was negatively correlated with TGAB in the non-metastatic group and positively correlated with TSH in the metastatic group.

In the metastatic group, TG levels were positively correlated with TSH levels (*r* = 0.253, *P* = 0.003); TG showed no correlation with CEA, CT, or TGAB (*r*=−0.114, *P* = 0.186; *r*=−0.168, *P* = 0.051; *r* = 0.037, *P* = 0.673). (Fig. 5).

### The diagnostic value of various variables in LNM of PTMC patients

The grouping of postoperative pathological results (metastatic group = 1, non-metastatic group = 0) was defined as the dependent variable, and the Nominal diameter, Gender, ALB, APOB, and TG were defined as the independent variables. A binary logistic regression analysis was performed to determine the combined predictive factors of nodal diameter (X1), gender (X2), ALB (X3), APOB (X4), and TG (X5). The regression equation is Y = 1.950 + 2.079 × 1 + 1.430 × 2-0.069.069 × 3-2.266.266 × 4 + 0.017 × 5, and the joint prediction factor is used as the five indicators combination to analyze the results.

**Fig. 6:**
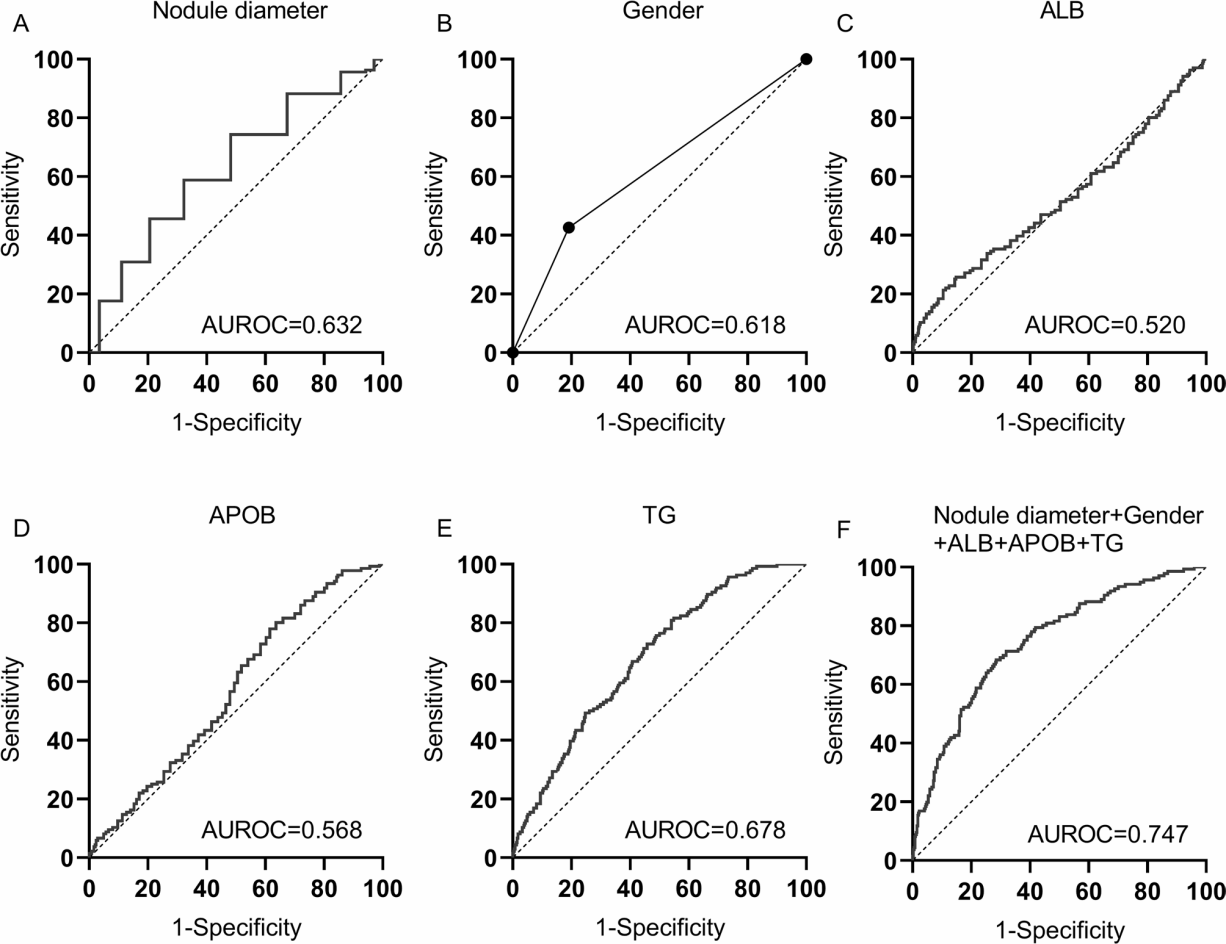
ROC curves of each indicator in the diagnosis of LNM in PTMC patients. **A**. ROC curves of nodal diameter. **B**. ROC curves of gender. **C**. ROC curves of ALB. **D**. ROC curves of APOB. **E**. ROC curves of TG. **F**. ROC curves of five indicators combination. The combined model showed higher AUROC (0.747) than single predictors.

The ROC curves were drawn using five variables (nodal diameter, gender, ALB, APOB, TG) and five indicators combination (nodal diameter + gender + ALB + APOB + TG) identified by LASSO-Logistic regression analysis, as shown in Fig. 6 (A-F). When the AUROC value detected by five indicators combination (nodal diameter + gender + ALB + APOB + TG) is 0.747, and the cutoff value is > 0.26642, the sensitivity and specificity are 68.38% and 71.43% respectively, and the PPV and NPV are 45.40% and 86.70% respectively. (Table 4).

**Table 4:** The diagnosis value of each indicator for LNM in PTMC patients.

Indicator	Youden index	Cutoff	AUROC	sensitivity	Specificity	AUROC 95%CI	PPV(%)	NPV(%)
Nodal diameter	0.2030	> 0.66	0.632	58.82	61.48	0.589–0.673	34.6	81.1
Gender	0.2351	male	0.618	42.65	80.87	0.575–0.659	43.6	80.3
ALB	0.1094	≤ 42.1	0.520	25.74	85.20	0.476–0.563	37.6	76.8
APOB	0.1570	≤ 0.99	0.568	77.94	37.76	0.525–0.611	30.3	83.1
TG	0.2688	> 8.75	0.678	72.79	54.08	0.637–0.718	35.5	85.1
Five indicators combination	0.40	> 0.26642	0.747	68.38	71.43	0.707–0.783	45.40	86.70

Note: PPV = positive predictive value, NPV = negative predictive value.

The data in Table 4 indicates that the AUROC values for the five variables (nodal diameter, gender, ALB, APOB, and TG) identified through LASSO-logistic regression analysis were each below 0.7, which had average diagnostic performance. The AUROC value of the combined test of the five indicators was 0.747, with good diagnostic performance. When the AUROC of each indicator was compared using medcalc software, the AUROC value of the five indicators combination (nodal diameter + gender + ALB + APOB + TG) was higher than the AUROC of each indicator individually, and the difference was significant from a statistical standpoint (*p* < 0.05), as shown in Table 5.

**Table 5:** Comparison of AUROC area of each indicator and five indicators combination.

Detection indicators	Z value	*P* value
Nodal diameter vs. Gender	0.389	0.697
Nodal diameter vs. ALB	2.719	0.007
Nodal diameter vs. APOB	1.577	0.115
Nodal diameter vs. TG	1.442	0.149
Nodal diameter vs. five indicators combination	4.423	< 0.001
Gender vs. ALB	2.346	0.019
Gender vs. APOB	1.227	0.220
Gender vs. TG	1.668	0.095
Gender vs. five indicators combination	5.713	< 0.001
ALB vs. APOB	1.313	0.189
ALB vs. TG	3.987	< 0.001
ALB vs. five indicators combination	6.134	< 0.001
APOB vs. TG	3.141	0.002
APOB vs. five indicators combination	5.553	< 0.001
TG vs. five indicators combination	2.388	0.017

### Precision recall curves for each indicator and five indicators combination

**Fig. 7:**
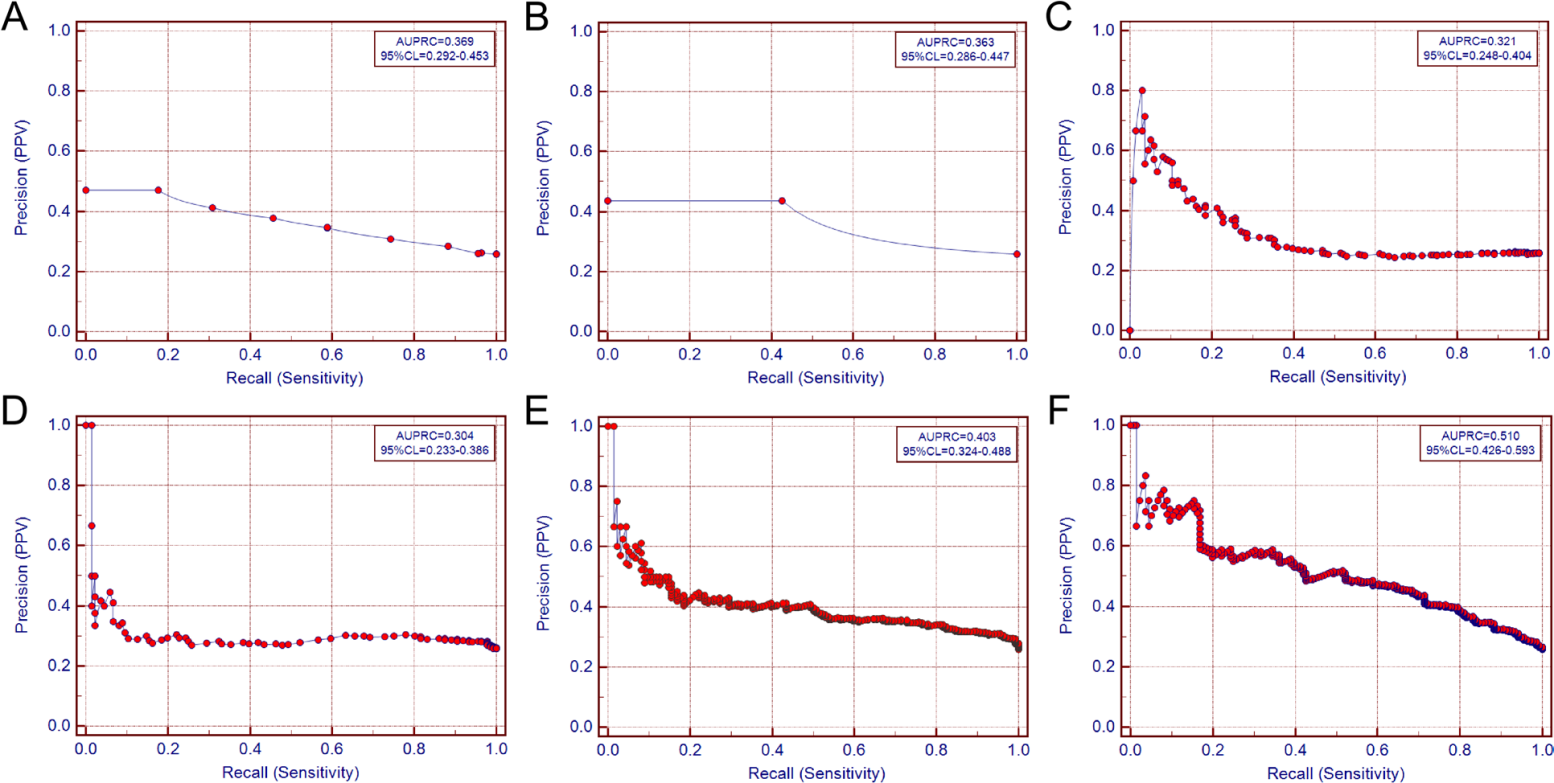
Precision-recall curves of each indicator and five indicators combined test. (**A**: precision-recall curves of nodal diameter; **B**: precision-recall curves of gender; **C**: precision-recall curves of ALB; **D**: precision-recall curves of APOB precision-recall curves; **E**: TG’s precision-recall curves; **F**: five indicators combination precision-recall curves. The combined model had the highest AUPRC (0.510), outperforming individual predictors, the horizontal coordinate X is Recall, the true positive rate; the vertical coordinate is Precision, the positive predictive value).

The ROC curves remain unchanged when the test set has a highly imbalanced positive and negative sample ratio, and their ability to discriminate does not depend on the ratio of positive to negative samples. This insensitivity, however, makes it hard to determine the prediction status of the model when the ratios of positive and negative samples change. PRC are more affected by the imbalance between positive and negative sample ratios, and can show the effect of the model with the change of the sample proportions, which can better reflect the actual effect and usefulness of the prediction model. The precision recall curves perform that AUPRC of Nodal diameter is 0.369(0.292–0.453); the AUPRC of Gender is 0.363(0.286–0.447); the AUPRC of ALB is 0.321(0.248–0.404); the AUPRC of APOB is 0.304(0.233–0.386); the AUPRC of TG is 0.403(0.324–0.488); the AUPRC of five indicators combination is 0.510(0.426–0.593) (Fig. 7). Compared to the single indicators, the combination test had a superior AUPRC, and the trend was basically consistent with the ROC curve evaluation results, indicating that five indicators combination had better classification performance.

### Clinical DCA for each indicator alone and for the five indicators combination

**Fig. 8:**
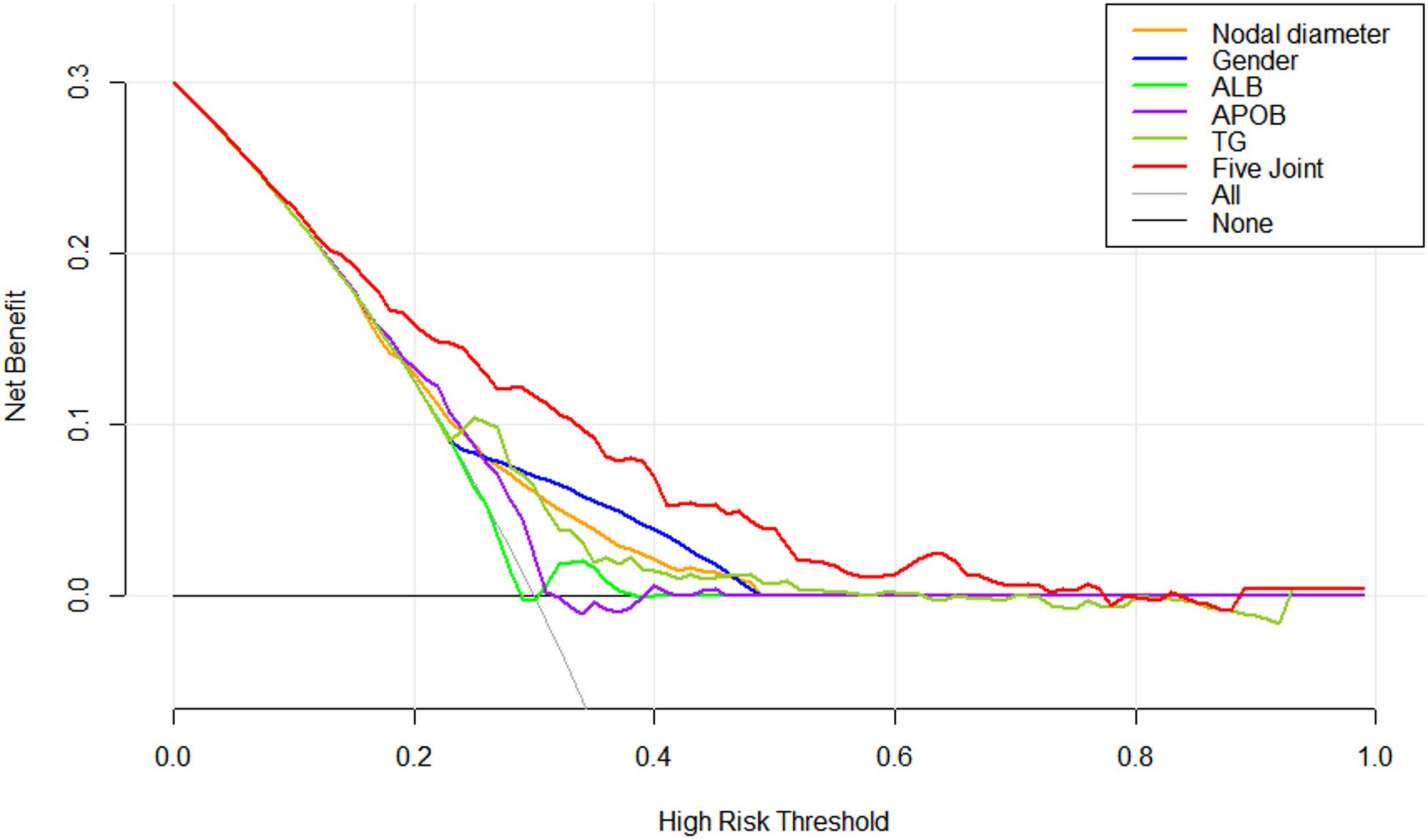
Clinical DCA for each indicator. The five-indicator model provided the greatest net benefit across threshold probabilities of 3–77%. (Note: The threshold probability is represented on the X-axis, and the net benefit is represented on the Y-axis, the black line labeled ‘None’ suggests that no PTMC patients have LNM, while the gray line labeled ‘AII’ indicates that all PTMC patients have LNM.).

The DCA for each indicator demonstrated that when the probability of nodal diameter threshold was 14%−48% for nodal diameter alone; 24%−48% for gender alone; 22%−39% for ALB alone; 10%−31% for APOB alone; 24%−62% for TG alone and at 3%−77% threshold probability for the five indicators combination, the net clinical benefit rate for each indicator was greater than that of the regimen in which all PTMC patients had lymph node metastases or none of them had metastases. The results indicate that the overall clinical benefit rate of the five-combination test was better than that of the single test (Fig. 8).

### Assessment of clinical impact value for each indicator based on DCA curve

**Fig. 9:**
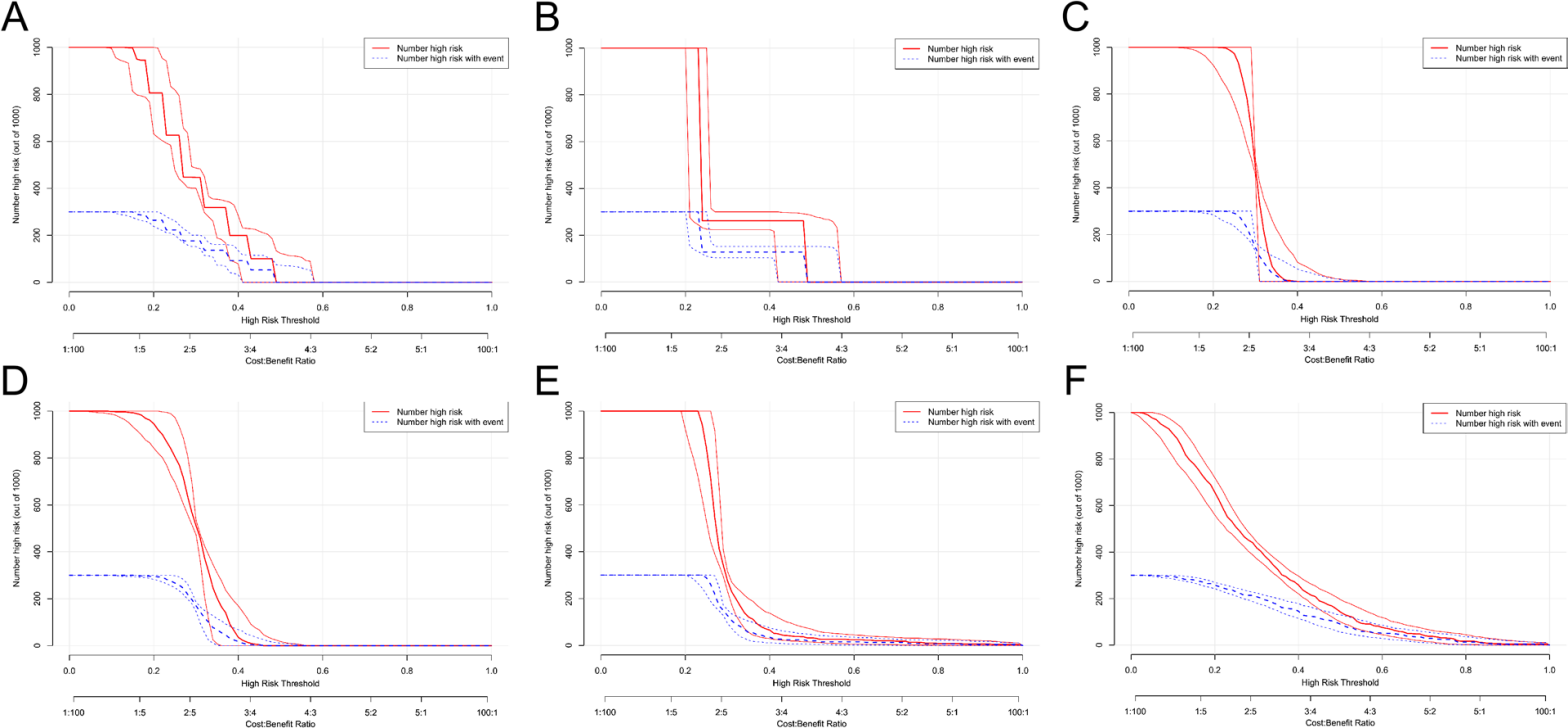
The DCA curve assesses the clinical impact value of each metric. **A**: CIC curve for nodal diameter; **B**: CIC curve for gender; **C**: CIC curve for ALB; **D**: CIC curve for APOB; **E**: CIC curve for TG; **F**: CIC curve for the combination of the five items. (Note: The threshold probability is plotted on the X-axis, and the Y-axis displays the number of high-risk cases from 1000 predictions. Red curves show predicted positives with 95% CI, blue curves show true positives with 95% CI. Predicted and observed values aligned closely, supporting clinical applicability).

The CIC of the model was plotted using the plot_clinical_impact() function in R software. The model was applied to forecast risk stratification for a group of 1000 individuals, illustrating the ‘loss’ and ‘benefit’ dimensions, with 10 levels assigned and confidence intervals shown. Figure 9 (A-F) exhibited CIC for each indicator and the five indicators combination, with the red curves show the number of predicted positive cases and 95% CL for each threshold probability, and the blue curves show the number of true positive cases and 95% CL for each threshold probability. These suggested that as the threshold probability increases, the predicted positive cases for each indicator closely match the actual number of cases, indicating that each indicator has certain clinical impact value.

### Nodule diameter, Gender, ALB, APOB, TG predict the risk of LNM in PTMC patients

**Fig. 10:**
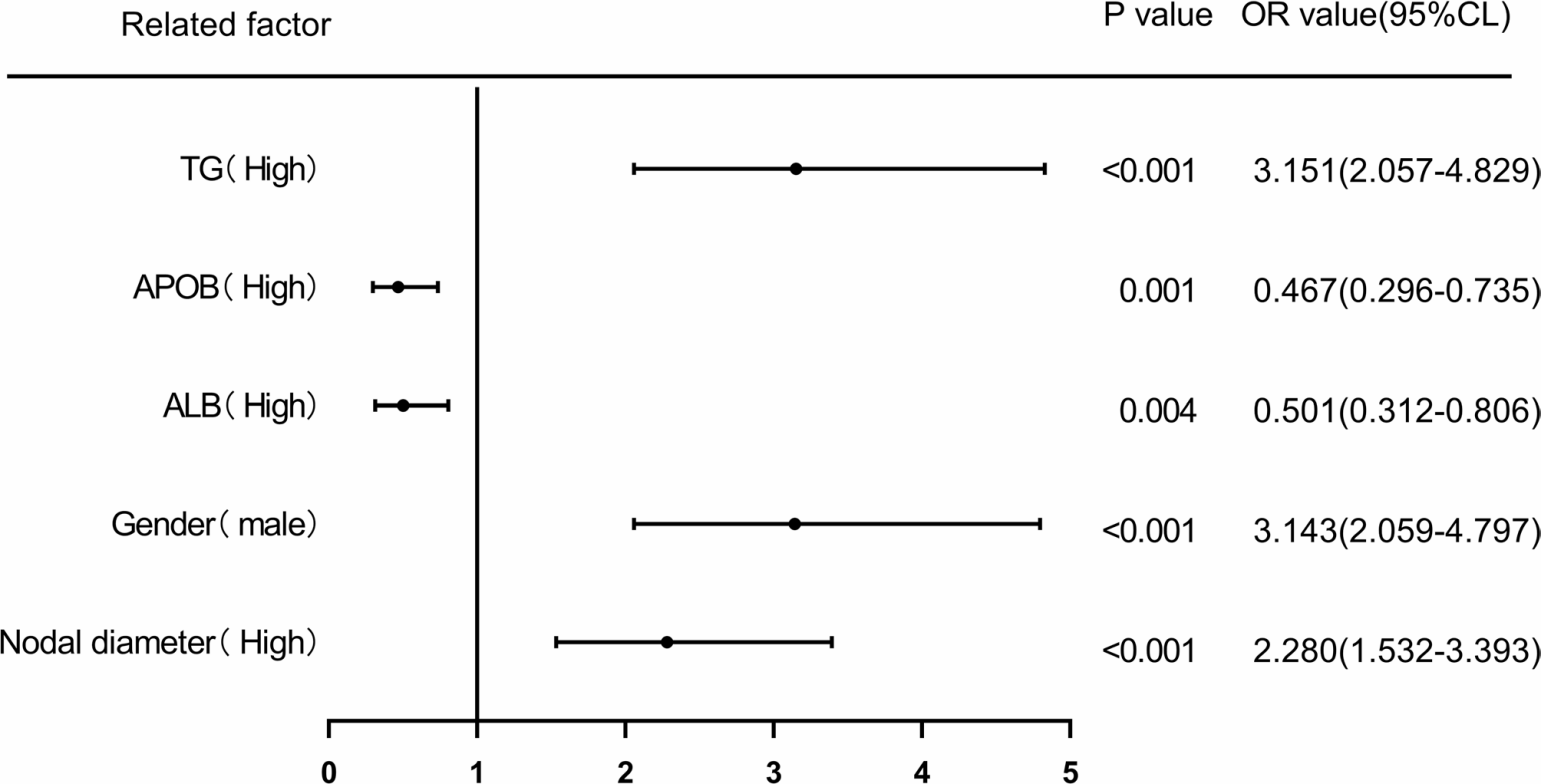
Forest chart of univariable logistic regression analysis of nodal diameter, gender, ALB, APOB and TG in predicting LNM in PTMC patients. Odds ratios with 95% CIs are shown. Larger nodal diameter, male gender, and higher TG increased risk; higher ALB and APOB were protective.

**Fig. 11:**
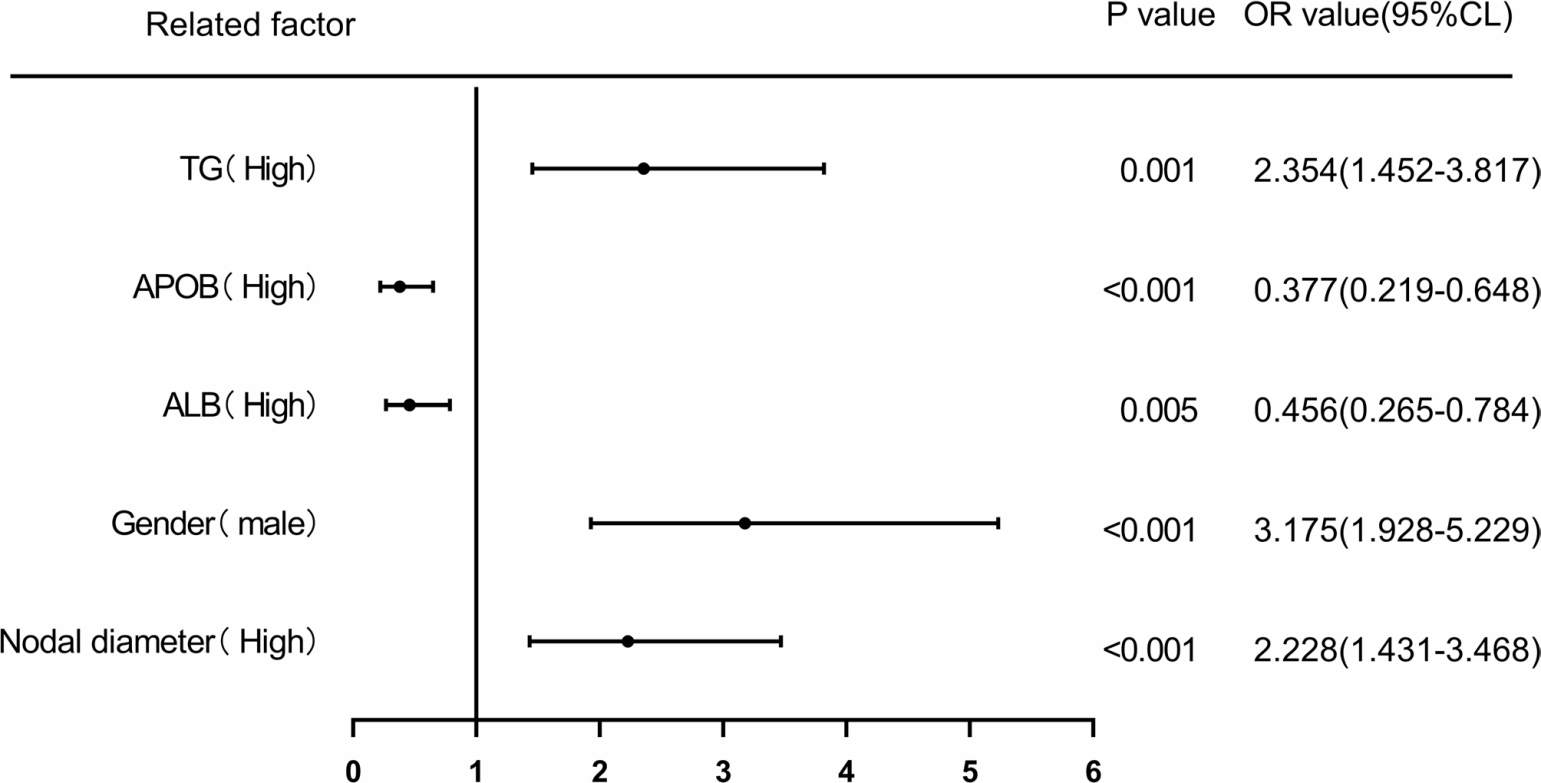
Forest chart of multivariable logistic regression analysis of nodal diameter, gender, ALB, APOB and TG in predicting LNM in PTMC patients. Male gender, larger nodal diameter, and higher TG were independent risk factors; higher ALB and APOB remained protective. (Note: multivariable correction of included variables nodal diameter, gender, ALB, APOB, TG, age, WBC, TGAB, TPO).

NBinary logistic regression analysis was used to assess the risk of LNM in PTMC patients by evaluating nodal diameter, gender, ALB, APOB, and TG. The cutoff value calculated from the ROC curve was used as the cutoff point to classify the two groups (high level group and low level group), and the levels of nodal diameter (> 0.66), Gender (male), ALB (> 42.1), APOB (> 0.99), and TG (> 8.75) were classified as high level group. The OR for the risk of LNM in patients with high levels of nodal diameter was 2.280 (95% CI: 1.532–3.393) compared to patients with low levels of nodal diameter (*p <* 0.001), while the corrected OR was 2.228 (95% CI:1.431–3.468) (*p <* 0.001); the OR for the risk of LNM in male patients compared with female patients was 3.143 (95% CI:2.059–4.797) (*p <* 0.001), while the corrected OR was 3.175 (95% CI:1.928–5.229) (*p <* 0.001); the OR for the risk of LNM in patients with high ALB compared with patients with low ALB was The OR for the risk of LNM was 0.501 (95% CI: 0.312–0.806), compared with patients with low-level ALB (*p <* 0.05), with a concurrent corrected OR of 0.456 (95% CI: 0.265–0.784) (*p <* 0.05); the OR for the risk of LNM in patients with high-level APOB compared with patients with low-level APOB was 0.467 (95% CI: 0.296–0.735) compared to patients with low-level APOB (*p <* 0.05), with a concurrent corrected OR of 0.377 (95% CI, 0.219–0.648) (*p <* 0.001); and an OR of 3.151 (95% CI, 2.057–4.829) for the risk of LNM in patients with high-level TG compared to patients with low-level TG (*p <* 0.001), while the corrected OR was 2.354 (95% CI :1.452–3.817) (*p <* 0.05). (Figures 10 and 11).

### Production of nomogram plot for predicting the risk of LNM in PTMC patients based on nodal diameter, gender, ALB, APOB, and TG

**Fig. 12:**
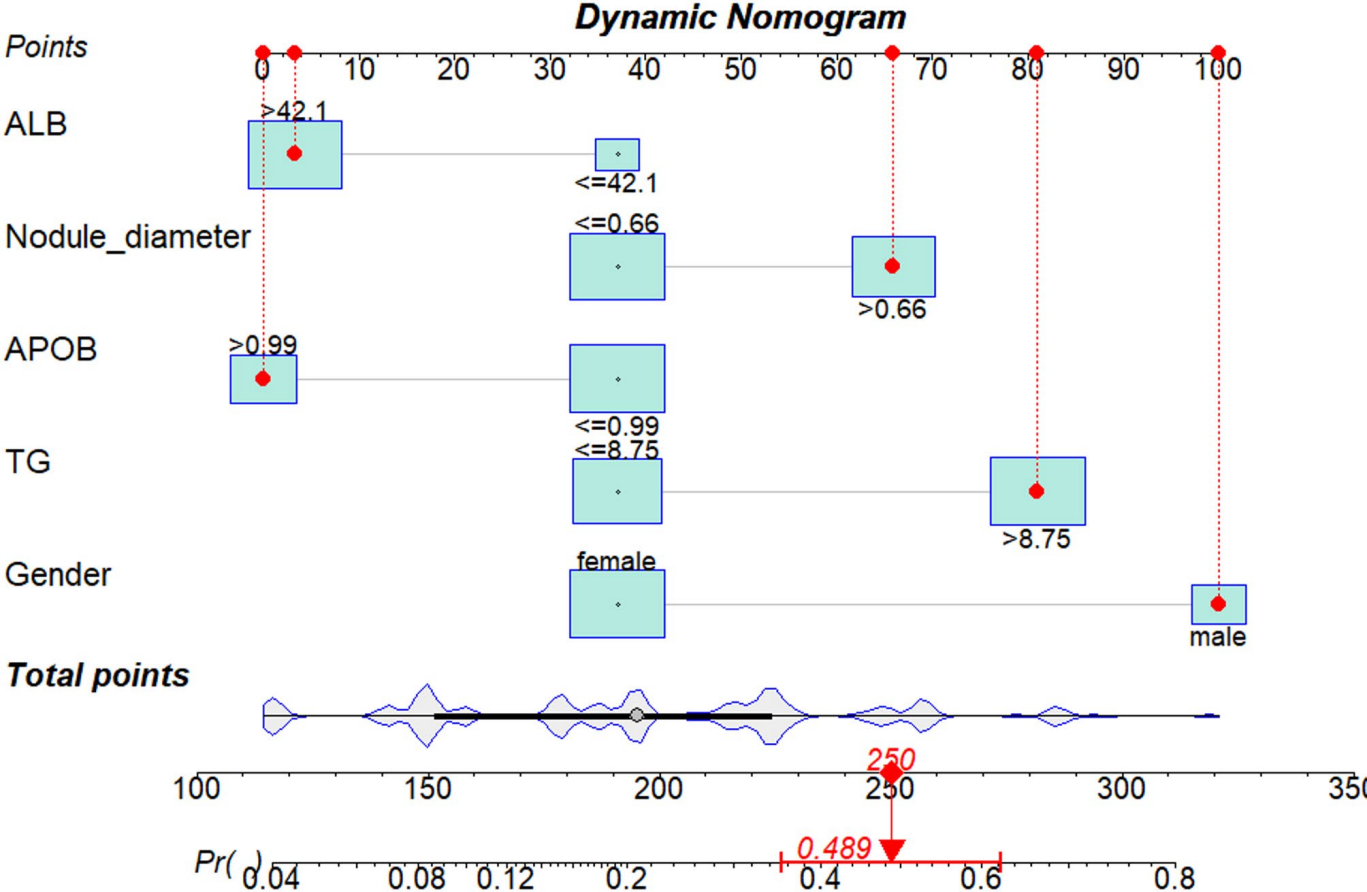
Nomogram plot for predicting LNM in PTMC patients. Total score corresponds to probability of LNM. Example shown indicates 250 points equating to an estimated 49% risk.

The multivariable analysis was employed to screen the meaningful variables nodal diameter, gender, ALB, APOB, and TG to include them in the prediction model. A threshold for the variable was established based on the cutoff value derived from the ROC curve, and after dichotomous transformation, a dynamic nomogram was constructed, as shown in Fig. 12. The logistic regression parameters of each variable in the nomogram model are shown in Table 6.

The corresponding scores in the nomogram are as follows: nodule diameter ≤ 0.66 is 37 points, nodule diameter > 0.66 is 66 points; APOB ≤ 0.99 is 37 points, APOB > 0.99 is 0 points; TG ≤ 8.75 is 37 points, TG > 8.75 is 81 points; gender as female is 37 points, gender as male is 100 points; ALB ≤ 42.1 is 37 points, ALB > 42.1 is 3 points. For PTMC patients, the probabilities of LNM corresponding to total scores of 50, 100, 150, 200, 250, 300, 350, and 400 are 0.009, 0.028, 0.084, 0.228, 0.487, 0.754, 0.908, and 0.970, respectively. As shown in the figure, when a male subject has ALB > 42.1, nodule diameter > 0.66, APOB > 0.99, TG > 8.75. Each variable is marked with a red dot on the graph and connected to its corresponding score with a red dashed line. By summing the scores corresponding to each variable, a total of 250 points is obtained, indicating a LNM risk of 0.489 for the observed subject. At the same time, to simplify the model application, we developed a web calculator, URL: https://ley120.shinyapps.io/Lymph_Node_Metastasis_in_PTMC/. To aid clinical staff in assessing the risk of LNM in PTMC patients by inputting diverse variable indicators.

**Table 6:** Logistic regression parameters of variables in the nomogram model.

Variables	B	S.E.	*P* value	OR	95%CI	
					Lower limit	Upper limit
(Intercept)	−1.568	0.305	0.000	0.209	0.113	0.374
Nodule_diameter > 0.66	0.676	0.222	0.002	1.966	1.274	3.050
APOB > 0.99	−0.868	0.260	0.001	0.420	0.248	0.691
ALB > 42.1	−0.790	0.268	0.003	0.454	0.269	0.769
TG > 8.75	1.029	0.237	0.000	2.799	1.771	4.499
Gender(male)	1.475	0.244	0.000	4.369	2.721	7.096

### Validation of the prediction model for the risk of LNM in patients with PTMC

**Fig. 13:**
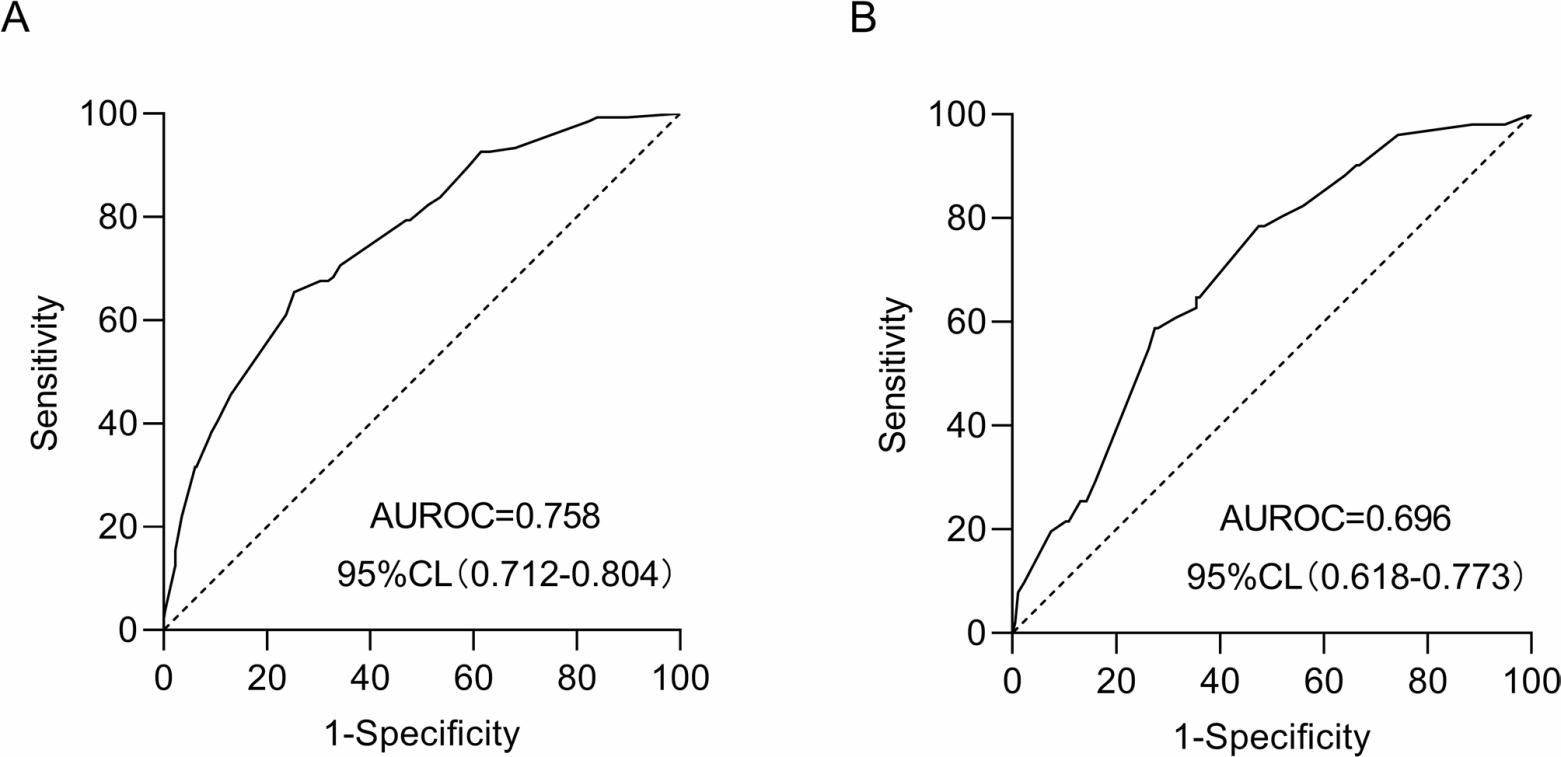
ROC curve to assess the predictive model of LNM risk in PTMC patients. **A**: ROC curve of the predictive model in the modeling set; **B**: ROC curve of the prediction model on the validation set.

**Fig. 14:**
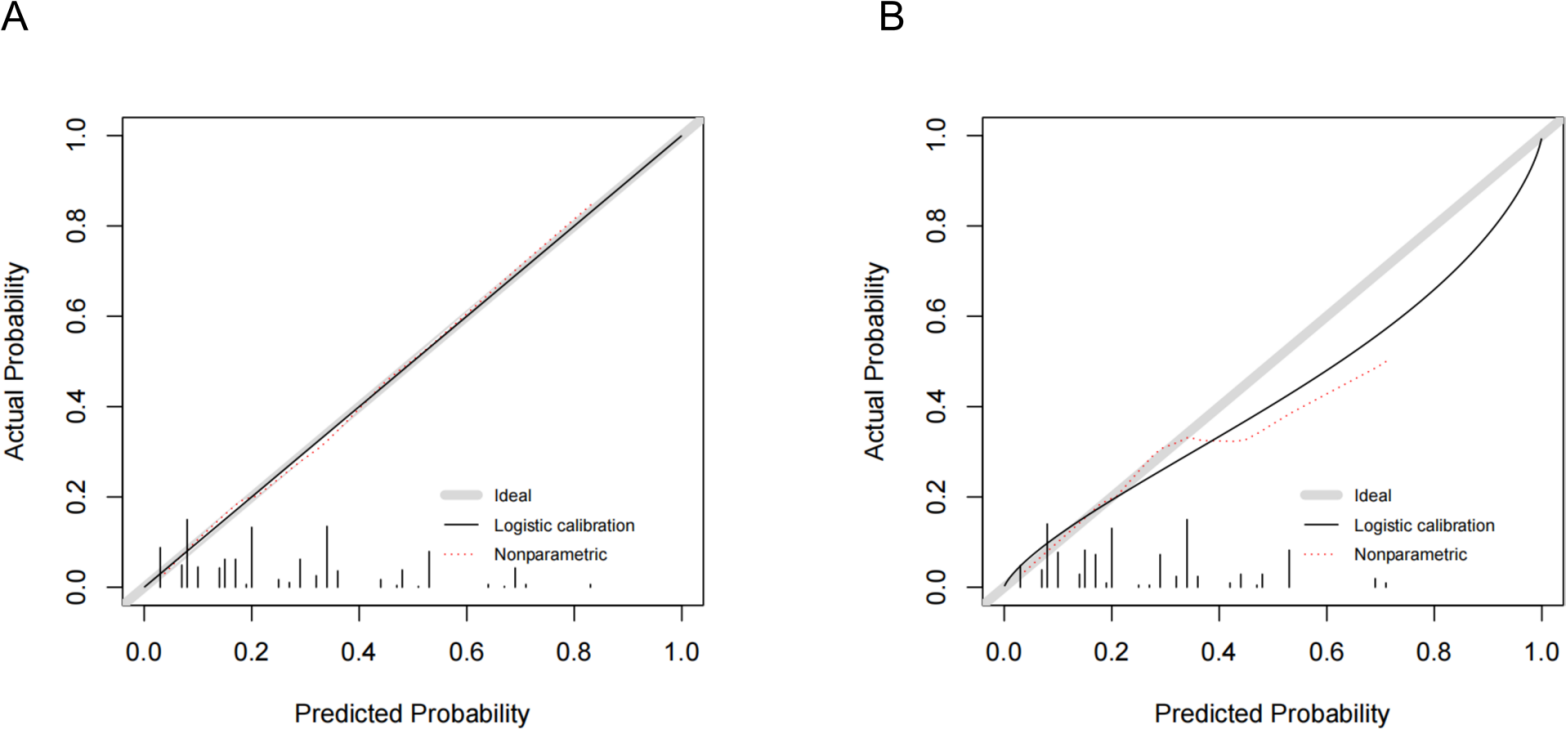
Calibration curve predictive model to assess the risk of LNM in patients with PTMC. Predicted probabilities were consistent with observed outcomes in both modeling and validation sets. **A**: Calibration curve of the prediction model in the modeling set. **B**: Calibration curve of the prediction model on the validation set.

The models were evaluated using discrimination and calibration, and discrimination was evaluated by calculating AUROC results for the predicted probability. The AUROC value for the modeling set model was 0.758 (0.719–0.804) and the AUROC value for the validation set model was 0.696 (0.618–0.773), indicating that the prediction model has favorable discrimination (Fig. 13A-B). The calibration degree of the prediction model refers to the consistency of the predicted probability with the actual observed value, the calibration curve and the calculation of the C-index, along with the Hosmer-Lemeshow test, illustrate the calibration degree. For the modeling set, the Hosmer-Lemeshow test results suggested no statistically significant difference between the predicted and actual risk values (χ^2^ = 9.069, *p* = 0.431), and the C-index value was 0.758 (95% CI: 0.712–0.803). For the validation set, the Hosmer-Lemeshow test showed that the difference between predicted and actual observations was not statistically significant (χ^2^ = 7.425, *p* = 0.593), and the C-index value was 0.696 (95% CI: 0.619–0.773). The modeling and validation sets had their calibration curves plotted, as illustrated in Fig. 14A-B. The results revealed that the model’s predicted probabilities are consistent with the actual probabilities. Additionally, to mitigate the risk of model overfitting, 10-fold cross-validation and Bootstrap resampling were employed on the modeling set. The AUROC for each fold, overall, and average AUROC along with the 95% CI were calculated and reported, as shown in Supplementary Table S1, The results showed an overall AUROC value of 0.758 (0.712–0.804) and an average AUROC value of 0.740 (0.698–0.786), indicating good model stability and limited risk of overfitting. The calibration curve was validated using the Bootstrap method with 1,000 internal re-samplings, as shown in Supplementary Figure S1. The mean absolute error was 0.01, the mean squared error was 0.00012, and the 0.9 quantile of absolute error was 0.016. These results demonstrated a good agreement between the predicted probabilities and the actual probabilities.

### Clinical DCA of the modeling set and validation set models

**Fig. 15:**
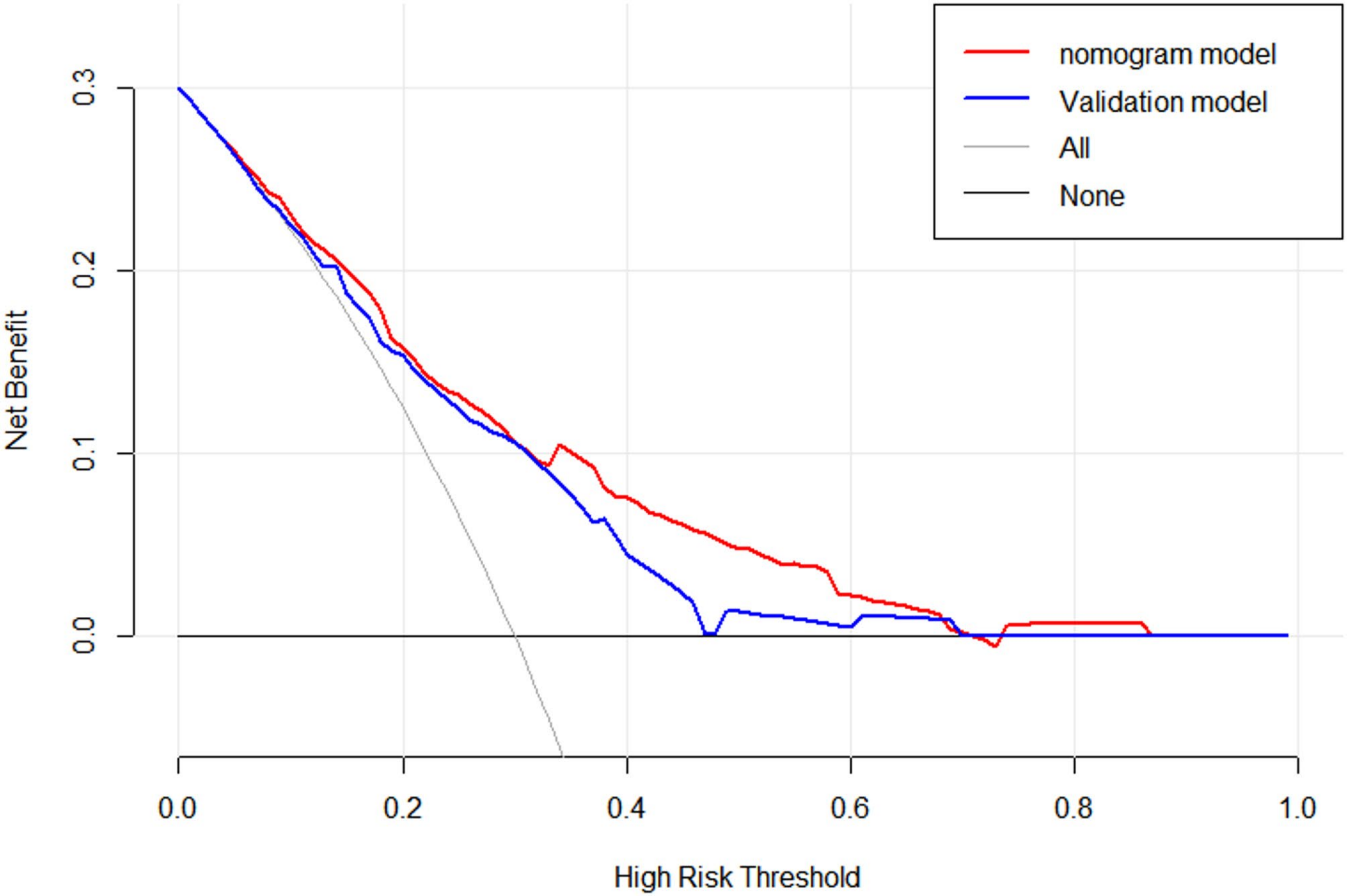
Clinical decision curves for the prediction model of risk of LNM in PTMC patients (Note: The threshold probability is represented on the X-axis, and the net benefit is represented on the Y-axis, the black line labeled ‘None’ suggests that no PTMC patients have LNM, while the gray line labeled ‘AII’ indicates that all PTMC patients have LNM).

The clinical DCA for each indicator showed that, when the threshold probability of the nomogram model is 0.05–0.71 and 0.74–0.86, when the threshold probability of the validation set is between 0.07 and 0.69, the clinical net benefit rate of the predictive model was greater than the scenarios where all PTMC patients either had LNM or did not have LNM. The results indicate a better overall clinical benefit rate for the prediction model, as shown in Fig. 15.

### Assessment of the clinical impact value for the model based on the modeling set and validation set model DCA curves

**Fig. 16:**
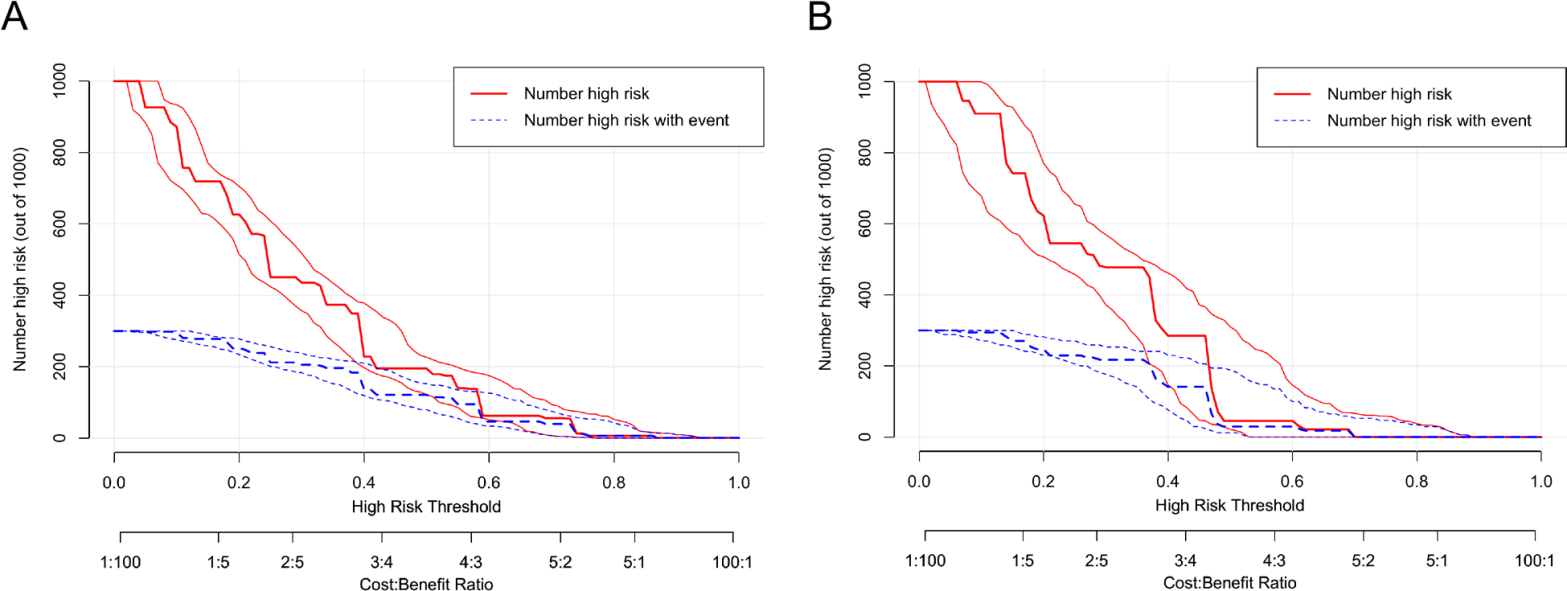
Assessment of the clinical impact value for the model based on the modeling set and validation set model DCA curves. **A**: CIC curve for the modeling set; **B**: CIC curve for the validation set; (Note: The threshold probability is plotted on the X-axis, and the Y-axis displays the number of high-risk cases from 1000 predictions. Red curves show predicted positives with 95% CI, blue curves show true positives with 95% CI. Predicted and observed values aligned closely, supporting clinical applicability.).

The CIC of the model was plotted using the plot_clinical_impact() function of R software. The model is used to predict risk stratification for 1000 people, showing the “loss” and “benefit” axes, with 10 levels assigned and confidence intervals shown, as shown in Fig. 16 (A-B) for the CIC for the modeled and validated sets of models, respectively. The red curves show the number of predicted positive cases and 95% CI for each threshold probability, and the blue or green curves show the number of positive cases and 95% CI for each threshold probability, CIC performed that as the threshold probability increases, the actual number of cases is similar to the number of positive cases predicted by the model, indicating that the model has a constructive clinical impact value. From a probabilistic perspective, patients with a model-predicted decision threshold of 0.4–0.7 can achieve clinical net benefit.

## Discussion

At the initial visit, tests for liver, renal, and coagulation functions were conducted to assess the patient’s overall health and the safety of anesthesia. Additionally, non-routine examinations, including tumor markers, blood lipids, and thyroid antibodies, were performed to aid in patient decision-making and enhance diagnostic accuracy. This study aims to avoid overlooking any potentially relevant predictors, 66 clinical and laboratory indicators were collected from patients with PTMC, including nodule number, maximum nodule diameter, LNM status, ultrasound TI-RADS classification, age, gender, and hematological parameters. The remaining 65 variables were analyzed using LASSO regression and multivariable logistic regression. Ultimately, only a few variables contributing to the prediction are retained to reduce noise and the risk of overfitting. The analysis identified maximum nodule diameter, gender, ALB, APOB, and TG as independent predictors of LNM risk in PTMC.

Nodule diameter, ALB, APOB, TG, and common thyroid tumor markers differed significantly between the metastatic and non-metastatic groups, suggesting their potential value in assessing LNM risk in PTMC. ROC curve analysis showed that the combined five-indicator model (nodule diameter, Gender, ALB, APOB, TG) achieved the highest AUROC compared with individual variables, indicating superior diagnostic performance. Nodule diameter was an independent predictor of LNM, with optimal discrimination at a cutoff > 0.66 cm. This finding aligns with prior studies reporting tumor size as an independent risk factor for central LNM and aggressiveness in PTMC^35–37^. Male gender was also identified as an independent predictor, consistent with evidence linking male gender to higher recurrence risk^38–41^. ALB levels were negatively associated with LNM risk, suggesting a potential protective role, although mechanisms remain unclear^42–45^. APOB, previously implicated in several cancers^46–49^, was also negatively associated with LNM in PTMC. Elevated TG levels were confirmed as a risk factor, consistent with prior reports^50^. PRC analysis demonstrated that the five-indicator model had the highest AUPRC, consistent with ROC results, confirming superior classification performance. DCA indicated greater net clinical benefit of the combined model compared with treating all or none of the patients, across a threshold probability of 3%–77%. CIC further showed that predicted positive cases closely matched observed cases, supporting the strong clinical applicability of these indicators.

The risk of LNM in PTMC patients was assessed using binary logistic regression based on nodule diameter, gender, ALB, APOB, and TG levels. Cutoff points for high and low levels were determined using ROC curves: nodule diameter > 0.66 cm, male gender, ALB > 42.1 g/L, APOB > 0.99 g/L, and TG > 8.75 mmol/L were classified as high-level. Patients with high nodule diameter had a 2.228-fold higher risk of LNM. Previous studies^51–53^ also reported that larger nodules predict LNM in PTMC, consistent with our findings. Male patients had a significantly higher risk of LNM than female patients (OR 3.175, 95% CI: 1.928–5.229; *p* < 0.001). This aligns with earlier findings^54,55^, and confirms that male gender is an independent risk factor, warranting closer clinical assessment. High ALB levels were associated with a 0.456-fold risk of LNM, suggesting a protective effect. ALB reflects both inflammatory and nutritional status^56^. Low ALB is often linked to chronic inflammation, and the inflammatory microenvironment can promote tumor progression^57,58^. Thus, reduced ALB may contribute to tumor aggressiveness through inflammatory mechanisms. High APOB levels were associated with a 0.377-fold risk of LNM, indicating another protective role. Clinical studies^59^ have shown that APOB expression is linked to prognosis in liver cancer, where low levels are associated with invasion and metastasis. Further research indicates that APOB may regulate metastasis indirectly through tumor microenvironment pathways, including TGF-β, T-cell receptor, and leukocyte transendothelial migration^60^. Altered APOB expression may therefore shape immune cell infiltration and activity, creating conditions that either favor or hinder cancer dissemination. However, the specific mechanisms require further validation. High TG levels were associated with a 2.354-fold higher risk of LNM, supporting TG as an independent risk factor in PTMC. Studies^61^ suggest that elevated TG promotes cancer cell detachment and lymphatic spread by influencing cell adhesion and extracellular matrix degradation. The association between TG and LNM in PTMC may involve TG mutations, synergy with driver genes such as BRAF, formation of metastasis-related molecular subgroups, and regulation of pro-metastatic factors such as STC1^61–64^. Rui et al.^65^ also confirmed TG as an independent risk factor for LNM in papillary thyroid cancer, underscoring the need for vigilance in patients with elevated TG levels.

A nomogram predicting the risk of LNM in PTMC patients was constructed using nodule diameter, gender, ALB, APOB, and TG, providing a simple visual tool for risk assessment. The scores of the five variables were summed to generate a total score, which was then projected onto the probability line to estimate the likelihood of LNM. The AUROC of the nomogram was 0.758, while that of the validation set was 0.696, indicating a moderate discriminative ability. Calibration curves, along with the C-index and Hosmer–Lemeshow test, were used to evaluate both the training and validation sets. The results showed that the predicted risks were consistent with observed outcomes. DCA showed that both datasets achieved higher net clinical benefits compared with strategies assuming all or none of the patients had LNM. This benefit was observed when the threshold probabilities were 0.05–0.71 and 0.74–0.86 for the nomogram model, and 0.07–0.69 for the validation set. Both DCA and CIC analyses indicated improved net clinical benefit and clinical impact in the training and validation sets. Current studies^66–68^ on predicting LNM mainly rely on pathological features. However, as pathology results are typically obtained postoperatively, their clinical utility is limited. Compared with conventional predictors such as ultrasound features (e.g., morphology, margins, microcalcifications), tumor diameter, and BRAF mutation status, this model shows only moderate predictive power. However, because its indicators are inexpensive and easily accessible, it is particularly useful in settings with limited imaging resources or without access to genetic testing. It may serve as an auxiliary tool to identify patients who require additional surgery or are suitable for active surveillance. Furthermore, we believe that this model can serve as a complementary tool to ultrasound examination and FNAC in predicting the risk of LNM. This is particularly relevant in cases where ultrasound fails to definitively ascertain the malignancy of nodules. The model offers additional reference information that can enhance clinical decision-making.

Despite the potential utility of blood indicators in predicting LNM in PTMC, several limitations should be noted. First, this was a single-center retrospective study. Although strict inclusion and exclusion criteria were applied and internal validation methods (random split-sample, bootstrap resampling, and cross-validation) were used to reduce selection bias, the generalizability of the findings remains limited. Second, patients with autoimmune thyroiditis, diabetes, or other systemic diseases were excluded to minimize confounding, which may also reduce the representativeness of the study population. Third, a subset of patients with subcentimetric TI-RADS 1–4 nodules was included because they underwent surgery under special circumstances rather than standard indications. This may further limit the applicability of the model. Fourth, although many laboratory indicators were initially included, only five independent predictors remained after LASSO and multivariable regression, and the influence of unmeasured confounders cannot be completely excluded. Fifth, external multicenter validation was not performed. Future large-scale, prospective, multicenter studies are needed to confirm the robustness and generalizability of the model. Finally, the biological mechanisms underlying some predictors (e.g., apolipoprotein B and thyroglobulin) remain unclear and require further investigation.

In conclusion, PTMC patients’ LNM is independently predicted by nodal diameter, gender, ALB, APOB, and TG. We designed a web-based visual tool to calculate the risk of LNM in papillary thyroid microcarcinoma, based on these predictors, the model was created to help clinical staff predict the likelihood of LNM in PTMC patients by entering different variable indicators.

## Supplementary Information

Below is the link to the electronic supplementary material.

Supplementary Material 1

Supplementary Material 2

## Data Availability

All data generated or analyzed during the current study are available from the corresponding author on reasonable request. Corresponding author E-mail: shanganquan@tongji.edu.cn.

## Abbreviations

TI-RADS: Thyroid imaging reporting and data system
PTMC: Papillary thyroid microcarcinoma
ROC: Receiver Operating Characteristic
CIC: Clinical impact curves
BTM: Benign thyroid micronodules
ACR: American Radiological Society
NPV: Negative predictive value
PPV: Positive predictive value
AUC: Area under the curve
PRC: precision-recall curves
DCA: Decision curves
WBC: White blood cells
RBC: Red blood cells
HGB: Hemoglobin
MCV: Mean red blood cell volume
MCH: Mean red blood cell hemoglobin content
MCHC: Mean red blood cell hemoglobin concentration
PLT: Platelets
GLU: |Glucose
BUN: Urea nitrogen
CRE: Creatinine
UA: Uric acid
AST: Aspartate aminotransferase
ALT: Alanine aminotransferase
TP: Total protein
ALB: Albumin
GLOB: Globulin
TBIL: Total bilirubin
DBIL: Direct bilirubin
ALP: Alkaline phosphatase
GGT: Glutamyl transpeptidase
TRIG: Triglycerides
CHO: Cholesterol
HDL: High-density lipoprotein
LDL: Low-density lipoprotein
PT: Prothrombin time
INR: International normalized ratio
APTT: Activated partial thromboplastin time
FIB: Fibrinogen
TT: Thrombin time
DD: D-Dimer
T3: Triiodothyronine
T4: Thyroxine
TSH: Thyroid stimulating hormone
FT3: Free triiodothyronine
FT4: Free thyroxine
TPO: Thyroid peroxidase antibody
TGAB: Thyroglobulin antibody
CT: Calcitonin
AFP: A-fetoprotein
CEA: Carcinoembryonic antigen
TG: Thyroglobulin
