# Supplementary material for: Personalized prediction of lymph node metastasis in papillary thyroid microcarcinoma: a nomogram and web calculator
Source: Sci Rep. 2025 Nov 25;15:45288. doi: 10.1038/s41598-025-28483-8 (PMC12749263; doi:10.1038/s41598-025-28483-8)
Supplement: Supplementary file 1 — Supplementary Material 1 [file 41598_2025_28483_MOESM1_ESM.docx]

# Supplementary Table

Table S1. Discrimination performance of the predictive model by 10-fold cross-validation and bootstrap internal validation

| Fold | AUC | 95%CI_lower | 95%CI_upper |
| --- | --- | --- | --- |
| Fold_1 | 0.697 | 0.535 | 0.859 |
| Fold_2 | 0.703 | 0.562 | 0.843 |
| Fold_3 | 0.867 | 0.773 | 0.96 |
| Fold_4 | 0.666 | 0.508 | 0.823 |
| Fold_5 | 0.730 | 0.576 | 0.883 |
| Fold_6 | 0.821 | 0.697 | 0.945 |
| Fold_7 | 0.705 | 0.535 | 0.876 |
| Fold_8 | 0.837 | 0.708 | 0.965 |
| Fold_9 | 0.720 | 0.575 | 0.866 |
| Fold_10 | 0.658 | 0.472 | 0.843 |
| Overall | 0.758 | 0.712 | 0.804 |
| Mean | 0.740 | 0.698 | 0.786 |
