# Supplementary material for: Personalized prediction of lymph node metastasis in papillary thyroid microcarcinoma: a nomogram and web calculator
Source: Sci Rep. 2025 Nov 25;15:45288. doi: 10.1038/s41598-025-28483-8 (PMC12749263; doi:10.1038/s41598-025-28483-8)

Supplementary Figure

Figure S1. The calibration curve was validated using the Bootstrap method with 1,000 internal re-samplings


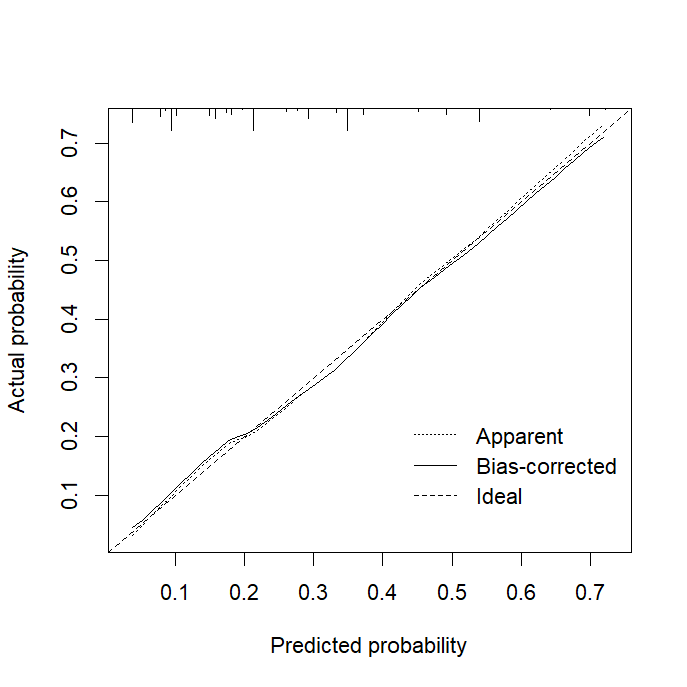

Supplement: Supplementary file 2 — Supplementary Material 2 [file 41598_2025_28483_MOESM2_ESM.docx]
